# Comparative Transcriptional Analyses of *Francisella tularensis* and *Francisella novicida*

**DOI:** 10.1371/journal.pone.0158631

**Published:** 2016-08-18

**Authors:** Siva T. Sarva, Robert H. Waldo, Robert J. Belland, Karl E. Klose

**Affiliations:** 1 University of Tennessee Health Science Center, Memphis, TN, United States of America; 2 South Texas Center for Emerging Infectious Diseases and Dept. of Biology, University of Texas San Antonio, San Antonio, TX, United States of America; New York Medical College, UNITED STATES

## Abstract

*Francisella tularensis* is composed of a number of subspecies with varied geographic distribution, host ranges, and virulence. In view of these marked differences, comparative functional genomics may elucidate some of the molecular mechanism(s) behind these differences. In this study a shared probe microarray was designed that could be used to compare the transcriptomes of *Francisella tularensis* subsp. *tularensis* Schu S4 (Ftt), *Francisella tularensis* subsp. *holarctica* OR960246 (Fth), *Francisella tularensis* subsp. *holarctica* LVS (LVS), and *Francisella novicida* U112 (Fn). To gain insight into expression differences that may be related to the differences in virulence of these subspecies, transcriptomes were measured from each strain grown *in vitro* under identical conditions, utilizing a shared probe microarray. The human avirulent Fn strain exhibited high levels of transcription of genes involved in general metabolism, which are pseudogenes in the human virulent Ftt and Fth strains, consistent with the process of genome decay in the virulent strains. Genes encoding an efflux system (*emrA2* cluster of genes), siderophore (*fsl* operon), acid phosphatase, LPS synthesis, polyamine synthesis, and citrulline ureidase were all highly expressed in Ftt when compared to Fn, suggesting that some of these may contribute to the relative high virulence of Ftt. Genes expressed at a higher level in Ftt when compared to the relatively less virulent Fth included genes encoding isochorismatases, cholylglycine hydrolase, polyamine synthesis, citrulline ureidase, Type IV pilus subunit, and the Francisella Pathogenicity Island protein PdpD. Fth and LVS had very few expression differences, consistent with the derivation of LVS from Fth. This study demonstrated that a shared probe microarray designed to detect transcripts in multiple species/subspecies of *Francisella* enabled comparative transcriptional analyses that may highlight critical differences that underlie the relative pathogenesis of these strains for humans. This strategy could be extended to other closely-related bacterial species for inter-strain and inter-species analyses.

## Introduction

*Francisella tularensis* is a highly virulent Gram negative bacterium. A pulmonary exposure to as few as 20 bacteria is believed to cause a fatal human disease [1]. Tularemia, the disease caused by *F*. *tularensis*, can present with different clinical symptoms depending on the route of entry into the human host. Ulcero-glandular tularemia is responsible for most of the natural cases of *F*. *tularensis* infection, but other clinical forms include occulo-glandular, gastrointestinal, and pulmonary tularemia. All forms can progress to a systemic infection that includes severe prostration, multi-system organ failure, and in some cases death. The threat of bioterrorism has sparked resurgence in research on this pathogen, in order to develop novel therapies and effective vaccine strategies [2, 3].

*F*. *tularensis* is classified further into several sub-species, including *F*. *tularensis* subsp. *tularensis* (Ftt), *F*. *tularensis* subsp. *holarctica* (Fth), and *F*. *tularensis* subsp. *mediasiatica* (Ftm). The bacterium *F*. *novicida* is alternately classified as a separate species, *F*. *novicida (Fn)* or as a Ft subspecies *F*.*tularensis subsp*. *novicida (Ftn)* [4]. While genomic relatedness might indicate that *novicida* should be classified as a Ft subspecies, arguments have been made to maintain these bacteria as a separate species, and we will utilize this separate species nomenclature here (Fn) [5]. An older classification system divided *F*. *tularensis* into highly virulent Type A strains, which now broadly corresponds to *Ftt*, and less virulent Type B strains, which correspond to *Fth* [6–8]. A live vaccine strain (LVS) was derived from Fth in the Soviet Union by repeated passage, and has been used to vaccinate humans. This strain is attenuated for virulence in humans but still can cause a lethal disease in mice. *Ftm* causes a mild disease and is geographically restricted to parts of Asia. *Fn* is considered non-pathogenic for healthy humans, but can cause a fatal disease in mice.

*F*. *tularensis* is known to infect a variety of cells such as macrophages, hepatic cells, endothelial cells, HeLa cells, mouse fibroblasts, and even amoebae [1, 9]. Its ability to infect and replicate in macrophages is critical for disease. The *Francisella* pathogenicity island (FPI), a cluster of genes that are essential for phagosome escape, intramacrophage replication, and virulence, encodes a Type VI secretion system [10–12]. There are two copies of the FPI in the *Ftt* and *Fth* strains, but only one copy in *Fn*. The FPI is virtually identical in the various *F*. *tularensis* strains, with the exception of the *pdpD* gene, which contains a deletion in Fth and a truncation in Ftt when compared to Fn *pdpD* [10]. The DNA binding protein PigR/FevR interacts with RNA polymerase complexed with the regulatory proteins MglA and SspA to activate transcription of the FPI genes[13–16]. Comprehensive transposon mutagenesis has identified additional genes that contribute to intra-macrophage survival, including genes involved in LPS O-antigen, capsule, siderophore, biotin, and DNA synthesis [17–23].

The first full genome annotation of *F*. *tularensis* to be published was that of the Ftt strain Schu S4 [24]. The genome is interspersed with multiple insertion sequences. The majority of insertion sequences, isftu1, belong to the IS630 Tc-1 Mariner class of transposases, while a smaller subset, isftu2, belong to the IS5 class. 30 percent of the annotated genes are hypothetical proteins, and a large number of these are unique to *F*. *tularensis*. 10 percent of coding sequences are predicted to be pseudogenes. The genome sequence of *Ftt* strain FSC198 differs from strain Schu S4 by only a few SNPs [25]. The genome sequence of *Ftt* strain WY96-3418 is also almost identical to Schu S4, but in contrast to FSC198, there is a marked difference in genomic organization [26]. These differences led to the division of Ftt strains into two clades: Type AI, which includes strains similar to strain Schu S4, and Type AII, which include strains similar to WY96-3418.

The genomes of *Fth* strains OSU18 [27], and FSC200 [28], differ by only a few SNPs. Fth shares extensive DNA sequence identity with Ftt, but shows a striking amount of genomic rearrangement in comparison with Ftt. Comparison of the genome of the Fth LVS strain with other Fth strains revealed 35 genes that have mutations predicted to alter protein sequence in LVS; some of these, including deletions in the genes encoding a Type IV pilus subunit (*pilA)* and an outermembrane protein (FTT0918), are likely the cause of virulence attenuation in this strain [28, 29]

The genome sequence of Fn strain U112 has given further insight into the evolution of *F*. *tularensis* [30]. It is clear that Fn, despite having ~98% identity at the nucleotide level with the more human virulent Ftt and Fth strains, contains only a fraction of pseudogenes compared to the other strains and has therefore more functional genes. It is hypothesized that the human pathogenic Ftt and Fth lineage emerged from a common ancestor with human non-pathogenic Fn, and during evolution, gene decay caused by IS transpositions and nucleotide substitutions led to increased pathoadaptation specific to human virulence in the Ftt and Fth strains [30].

In spite of the availability of large amounts of comparative genomics data, a comparative transcriptomics analysis for *F*. *tularensis* has not been published. The current study was designed to identify transcription differences that might contribute to differences between the virulence of the *F*. *tularensis* subspecies. The results discussed below give a summary of these findings.

## Methods

### Bacteria

*Ftt* strain Schu S4, *Fth* strains OR960246 and LVS, and *Fn* strain U112 were grown in Chamberlain’s medium [31] to an optical density of 0.6. The *Fth* strain OR960246 was obtained from the Centers for Disease Control; its genome was sequenced by Baylor Center for Bioinformatics and Computational Biology (https://wiki.umiacs.umd.edu/cbcb/index.php/Francisella_tularensis_holarctica_OR960246). OR960246 is the Fth strain we maintain in our laboratory and thus was chosen for analysis in the present study.

### Design of the Shared Probeset Microarray

**Fig 1:**
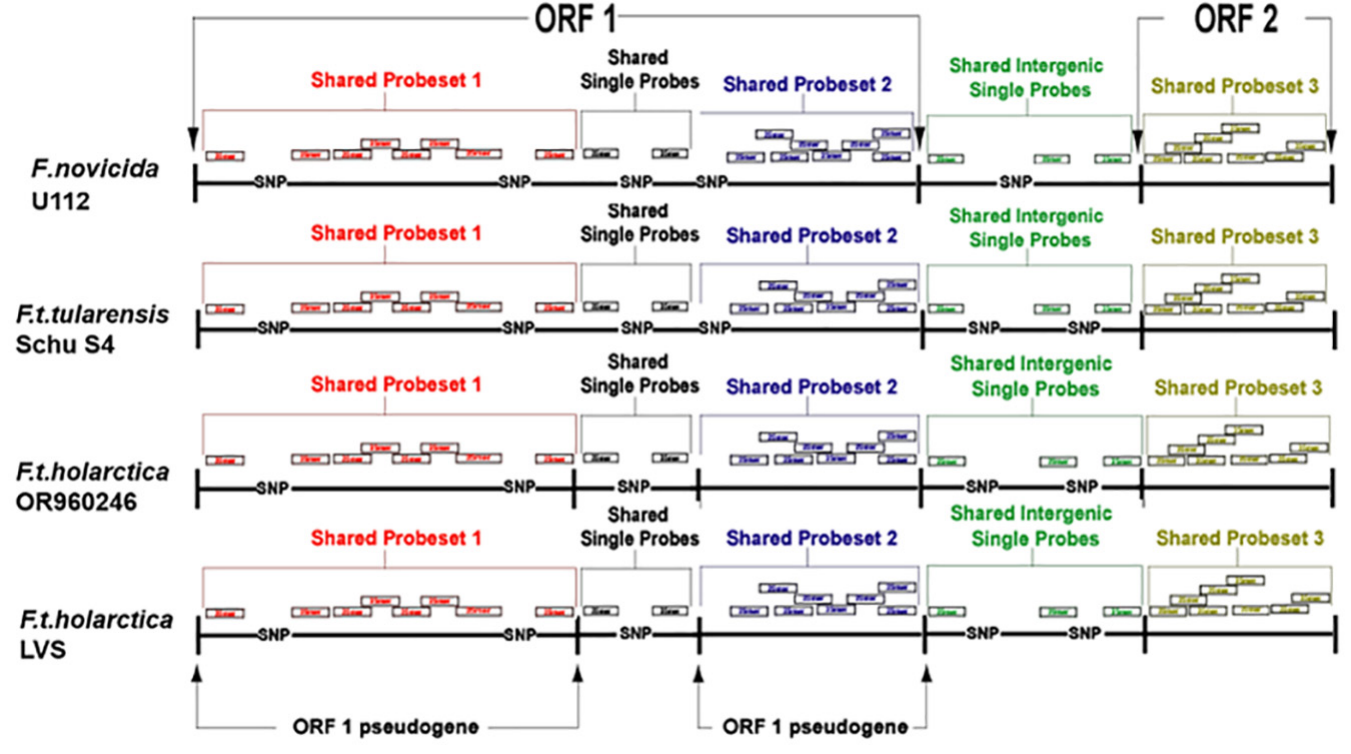
Design Summary of Multi Strain Shared Probe Sets. An example of how multiple shared probe sets were created in regions of ambiguity where a full length open reading frame (ORF) is present in one strain and the homologous ORF in the other strains is broken into fragments. In the hypothetical example shown, ORF1 is an ORF that is intact in *Fn* U112 and *Ftt* Schu S4, but the orthologs in both *Fth* strains are pseudogenes separated into two putative coding regions. Two probe sets were designed to cover each of the two fragments, such that the larger intact ORFs in Fn and Ftt were monitored by two probe sets, while each pseudogene in Fth was monitored by one probe set. The intergenic region between ORF1 and ORF2 also had shared probes. ORF2 is intact in all four strains so a single consensus probe set was designed. Probes were also designed for the anti-sense regions of the ORFs (not shown).

Fig 1 gives a simplified illustration of the design of the shared probe Nimble Express Affymetrix microarray. The four genome sequences used initially for the design of the microarray were Ftt strain Schu S4, Fth strains LVS and OSU18, and Fn strain U112 (AJ749949, AM233362, CP000439, and CP000437 respectively). Genome sequences of the four strains were formatted into strain specific raw sequence databases using “formatdb” of the NCBI tool box, and then aligned using megablast with the expectation value set to 1 x 10^−4^. Custom scripts were written to parse the output files to give alignment of genome sequences of the four strains. In places where more than one region of a target sequence could be aligned to an input sequence, the alignment that allowed the least amount of breaks in the raw sequences was selected.

Regions with a length of 25 nucleotides on both strands of the whole genome alignment were chosen for probe selection using custom scripts. If a dissimilar nucleotide was encountered in the alignment, a probe was not designed across the region of dissimilarity. By this methodology, consensus probes were designed throughout the length of all the four genomes. Every effort was made to design single shared probes for all the four strains; however if a single shared probe could not be designed that matched all four strains, then probes were designed that were shared for the maximum number of strains in the alignment, and strain-specific non-consensus probes were designed for the differing strains. Unique probes of 25 nucleotides were designed for the unique regions of each strain. This strategy resulted in complete coverage of the entire genome of all four strains, with a maximum gap in probe coverage of 10 nucleotides in the initial stage of design. The probe selection regions were extended up to a maximum of five nucleotides in either direction in some cases to allow for a selection of probes of better quality.

Next, due to the maximum probe set limit of the Nimble Express Affymetrix platform, steps were taken to reduce the number of probe sets that would need to be tiled on the microarray. In order to use similar annotation parameters for all four strains, Glimmer 2.0 was used to predict open reading frames (ORF) using default settings [32]. All ORFs with > 99% identity in all four strains and which had no paralogs in any of the strains were selected for “traditional” probe sets (11 probe pairs per gene: 11 match probes and 11 mis-match probes); 994 ORFs met these criteria. The “tiled” probe sets corresponding to insertion elements with a high copy number (isftu1 and isftu2) were removed and replaced by a single “traditional” probe set for isftu1; isftu2 was not included due to inability of the Affymetrix design pipeline to design a reliable probe set. The rest of the probes representing the remaining portions of the genomes were designed as single probe pair “tiled probe sets”. Seven additional sequences from plasmid pFNLTP, pFNLTP6_orf3, pFNLTP6_repA, pFNLTP6_orf2, pFNLTP6_kanR, pFNLTP6_ampR, and GFP, were selected for “traditional” probe sets. The sequences of the probe selection regions were subjected to hard pruning and soft pruning by the Affymetrix design team, to minimize cross-hybridization with host (human, mouse and rat) contaminant RNA.

The virulent *Fth* strain OR960246 was used for expression analysis. To maintain the shared probe set nature of the microarray, the draft genome sequence of OR960246 (Dr. Joseph Petrosino, Baylor College of Medicine; https://wiki.umiacs.umd.edu/cbcb/index.php/Francisella_tularensis_holarctica_OR960246) was used to identify the probes affected by subtle differences between OSU18 and OR960246, which were only a few single nucleotide polymorphisms (SNPs) and indels. The probes that were affected by these differences were corrected. Accession numbers of the orthologs from OSU18 are used to describe the corresponding ORFs of OR960246.

Microarrays were manufactured by Affymetrix using a custom manufacturing process. Two custom library files, one for monitoring ORF expression and another for monitoring intergenic expression, were designed in a format that is compatible with Gene chip operating system (GCOS). The library files were loaded into GCOS using the Library files update software provided by Affymetrix. The probe sequences, library files, and the complete design of the microarray were made public by submission to GEO database (GPL20119).

### Processing of Samples for Microarray Analysis

Ft strains LVS, Schu S4, and OR960246 and Fn strain U112 were grown to 0.6 optical density at 600 nm in Chamberlains defined medium (CDM) at 37°C [31]. RNA isolation, cDNA synthesis and microarray processing was performed as described previously [33, 34]. Total RNA was isolated using TRIZOL (Invitrogen) and treated with Turbo DNA-free DNase (Ambion). cDNA was synthesized and labeled according to the Affymetrix standard prokaryotic protocol. Labeled cDNA was hybridized to the custom Nimble Express Affymetrix microarray. All samples were performed in triplicate. GCOS (Affymetrix) was used to normalize the microarray data, setting the mean of the core probe sets (994) to 1000; the data was further analyzed using GeneSpring 7.2 and GeneSpring GX (Agilent Technologies). Genes with >/ = 5-fold difference in expression level between strains (p </ = 0.05) were selected for further analysis. The p-values were obtained using GeneSpring (One-Way ANOVA, parametric test assuming equal variance, Benjamin–Hochberg multiple testing corrections, no post-hoc testing). All of the raw data has been submitted to GEO database (GSE68478).

## Results and Discussion

### Shared Probeset Microarray

**Fig 2:**
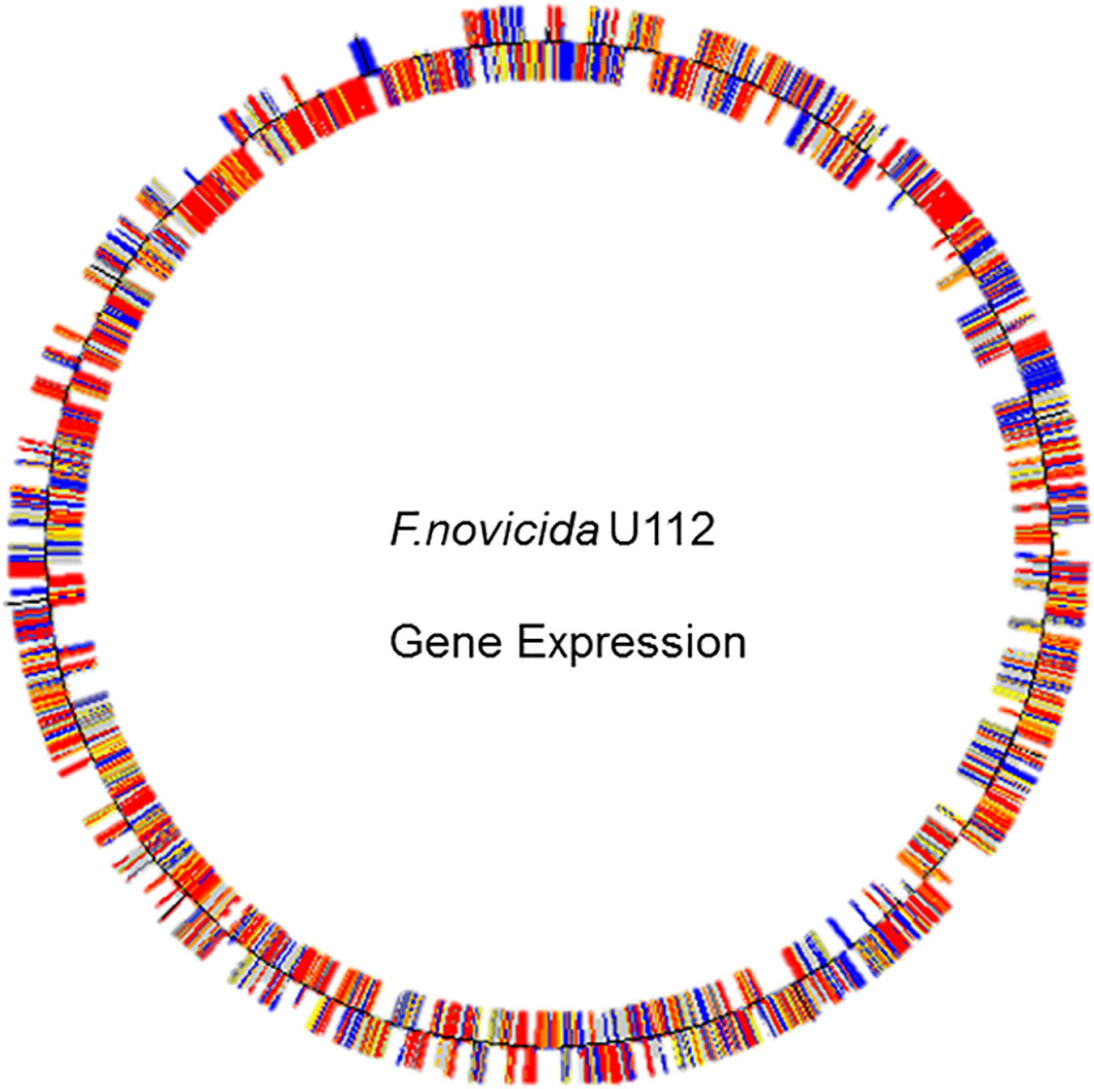
Expression of Open Reading Frames in *Fn* U112. Expression of predicted genes (open reading frames) in *Fn* U112 genome. Genes in the outer circle are on the plus strand while those in the inner circle are on the minus strand. Red indicates genes with high expression, yellow indicates genes with intermediate expression and blue indicates genes with low expression. The 12 O’clock position corresponds to the start of the genome.

We designed a shared probe *Francisella* whole genome microarray, utilizing four different genome sequences (Ftt strain Schu S4, Fth strains LVS and OSU18, and Fn strain U112). As described above, this microarray has a core set of shared probes to sequences that are common among the four strains (species/subspecies), as well as specific probes to those genes that are unique to a subset of these species/subspecies. Our goal was to create a single microarray for these species/subspecies that would enable comparative expression studies between Ftt, Fth, and/or Fn. All the strains in the study were grown to identical optical densities in Chamberlain’s medium (CDM), and their transcriptomes were analyzed by the shared probe microarray, as described above. Chamberlain’s medium was chosen due to the fact that it is the defined medium that can support growth of *Francisella* strains, and that we and others have already identified the proteome of various strains grown in this medium and can thus compare transcriptome to proteome [34]. An example of whole genome transcription in Fn strain U112 from this approach is shown in Fig 2, indicating that the expression of ORFs can be monitored using this microarray.

### Expression of Pseudogenes and Transposases in *Francisella*

The Ftt and Fth genomes contain a relatively large number of ORFs annotated as pseudogenes, when compared to the Fn genome, and this genomic decay has been ascribed to pathoadaptation by the more virulent strains [30]. Many of the ORFs annotated as pseudogenes in *Ftt*, *Fth* and *Fn* have high levels of transcription (S1, S2, S3, S4, S5 and S6 Tables). We previously observed that peptide fragments from pseudogenes are present in the *Ftt* proteome [34]. The biological significance of these transcribed and/or translated pseudogenes is not known.

The most prevalent insertion sequence (IS) element in the Ftt and Fth genomes is isftu1; *Ftt* strain Schu S4 has 50 copies, *Fth* strain OSU18 has 59 copies, whereas *Fn* strain U112 has only a single copy. Carlson *et al* showed that the transposase encoded in isftu1, along with that in another IS element isftu2, are transcribed in Ftt Schu S4 and Fth LVS, and their transcription increases in response to spermine [35]. Comparative transcriptional analysis performed with the shared probe microarray demonstrated that isftu1 was highly transcribed in both virulent sub-species (Ftt and Fth) during growth in CDM, in contrast to Fn, which showed no significant expression (S7 Table). Because all copies of isftu1 are identical, we could not determine if the high level of transcription is due to the cumulative effect resulting from the high copy number in Ftt/Fth, or if only a subset of isftu1 elements is transcribed to high levels. Transcription of the other prevalent IS element isftu2 could not be monitored, due to the lack of reliable probe sets on the microarray. The less prevalent IS elements, (isftu3, isftu4, isftu5, or isftu6) did not have comparable high expression levels in any of the strains. The high level transcription of the isftu1 transposase in Ftt and Fth suggests that these genomes have a potential for ongoing rearrangements.

### Differential RNA Expression in Strains of *Francisella*

**Fig 3:**
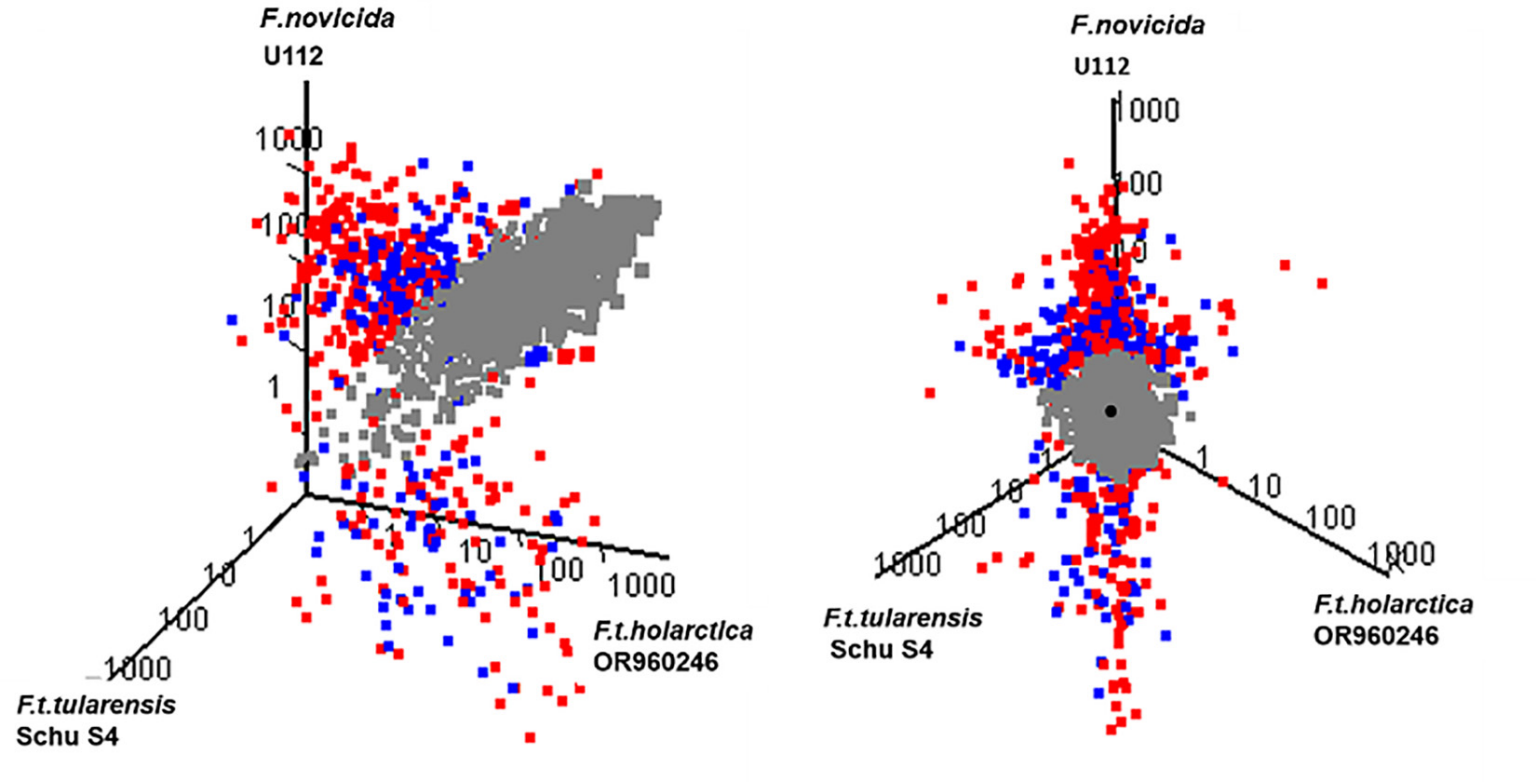
Comparison of Transcriptomes among Species/Subspecies of *F*.*tularensis*. The left and right panels show the same 3 dimensional graph visualized from two different angles. Each point represents a probe set monitoring the transcription of an ORF. The values on x, y and z axes represent the geometric mean of normalized intensity of the three biological replicates of *Ftt Schu S4*, *Fth OR960246 and Fn U112* respectively. Probe sets that have differential expression of less than five fold and/or a p-value greater than 0.05 in both of the one way comparisons of Ftt Schu S4 and Fth OR960246 with *Fn* U112 are colored grey. Of the remaining probe sets, those representing genes which are intact in all of the three strains are colored red, while the probe sets representing genes with at least one of the orthologs annotated as a pseudogene are colored blue.

Because the *Francisella* genomes largely share the same genes, and the microarray was designed to detect the shared gene set, transcription levels can be directly compared between the three *Francisella* species/subspecies grown under identical conditions. Interestingly, we found that transcription levels of many genes are notably different among the three species/subspecies of *Francisella* (Fig 3, Table 1, Table 2 and Table 3, S1, S2, S3, S4, S5 and S6 Tables). In general, transcription of relatively more genes was higher in Fn compared to Ftt or Fth. In addition to unique genes in each species/subspecies, pseudogene formation in *Ftt* and *Fth* due to possible genome decay may have contributed to significant transcriptional differences. The large number of pseudogenes in the Ftt and Fth genomes may be one cause of the difference in expression between Ftt/Fth and Fn, because the pseudogene may not be expressed to the same level as the corresponding gene in Fn, or the presence of a pseudogene may influence transcription of downstream genes. Likewise, transposons in Ftt and Fth may negatively impact transcription of genes by inserting into that gene or its promoter, or downstream genes within an operon. Finally genomic reorganization in Ftt and Fth may cause breaks within operons that place genes in a different genomic context. However, differential expression for a number of genes could not be easily attributed to one of these possibilities, and thus transcriptional differences between the strains may be due to differences in regulatory genes and/or networks elsewhere in the genome (i.e. *trans* effects).

**Table 1:** Genes with High Expression in *Ftt* Schu S4 Compared to *Fn* U112**.

Locus in Ftt	Locus in Fn	Fold Difference*	Product
FTT0027	FTN_1684	16	pyridoxal-dependent decarboxylase (lys1)
FTT0028	FTN_1683	6	drug:H+ antiporter-1 (DHA1) family protein
FTT0029	FTN_1682	17	siderophore biosynthesis protein (fslA)
FTT0103	FTN_1612	5	transposase
FTT0221	FTN_0090	5	acid phosphatase (precursor)(acpA)
FTT1140	FTN_1122	9	hypothetical protein
FTT1242	FTN_1260	10	hypothetical protein
FTT1653	FTN_0030	99	hypothetical protein
FTT1654	FTN_0029	190	HlyD family secretion protein(emrA2)
FTT1655	FTN_0028	72	hypothetical protein

*≥ 5 fold

p ≤ 0.05

** The table only lists the genes that are intact in both Ftt and Fn. Please see S1 and S2 Tables for a complete list that includes pseudogenes and missing genes, as well as genes expressed at lower levels in Ftt compared to Fn.

**Table 2:** Genes with High Expression in *Ftt* Schu S4 Compared to *Fth* OR960246**.

Locus in Ftt	Locus in Fth	Fold Difference*	Product
FTT0127	FTH_1591	5	major facilitator superfamily (MFS) transport protein
FTT0442	FTH_1570	8	major facilitator superfamily (MFS) transport protein
FTT0707	FTH_1479	6	nicotinamide mononucleotide transport (NMT) family protein
FTT0784	FTH_1400	6	hypothetical protein
FTT0815	FTH_1370	5	chitin binding protein
FTT1004	FTH_1172	6	DMT superfamily drug/metabolite transporter
FTT1089	FTH_1087	5	isochorismatase hydrolase family protein
FTT1090	FTH_1086	8	possible NMC family Nicotinamide mononucleotide uptake permease PnuC
FTT1175	FTH_0772	5	hypothetical protein
FTT1234	FTH_0713	18	choloylglycine hydrolase family protein
FTT1414	FTH_0648	7	hypothetical protein
FTT1784	FTH_1866	11	hypothetical protein

*≥ 5 fold

p ≤ 0.05

** The table only lists the genes that are intact in both Ftt and Fth. Please see S3 Table for a complete list that includes pseudogenes and missing genes.

**Table 3:** Genes with Low Expression in *Ftt* Schu S4 Compared to *Fth* OR960246**.

Locus in Ftt	Locus in Fth	Fold Difference*	Product
FTT0845	FTH_0338	9	hypothetical protein
FTT1143	FTH_0806	14	hypothetical protein
FTT0642	FTH_0895	6	acetolactate synthase small subunit
FTT0158	FTH_1669	9	hypothetical membrane protein

*≥ 5 fold

p ≤ 0.05

** The table only lists the genes that are intact in both Ftt and Fth. Please see S4 Table for a complete list that includes pseudogenes and missing genes.

### Comparison of transcription in Ftt and Fn

**Fig 4:**
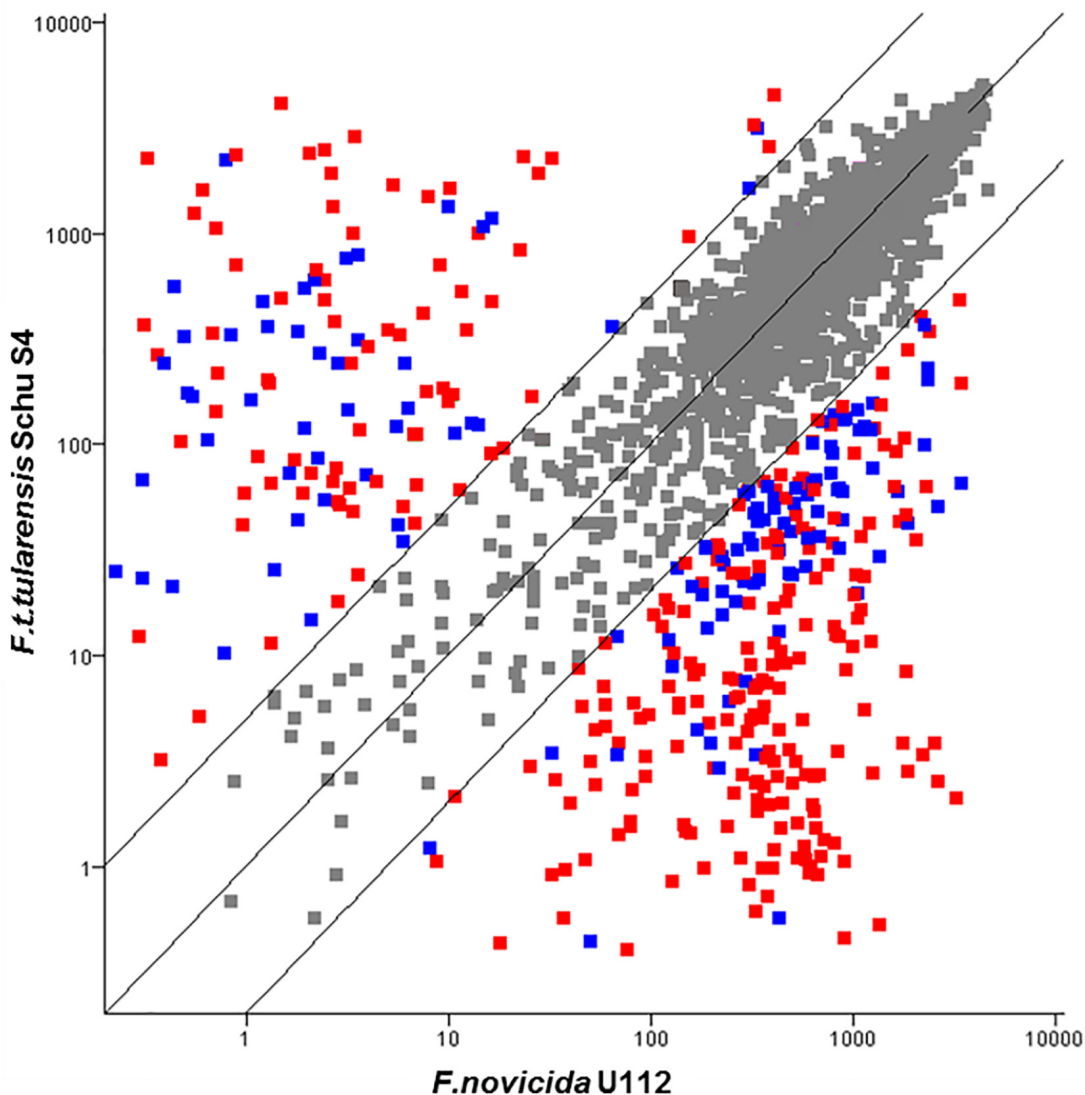
Comparison of Transcriptomes of *Ftt* Schu S4 and *Fn* U112. Despite high genomic identity, Ftt and Fn have large scale transcriptional differences when grown under identical conditions. Each point represents a probe set monitoring the transcription of an ORF. The values on x and y axes represent the geometric mean of three biological replicates. Probe sets that have differential expression of less than five fold and/or a p-value greater than 0.05 in one way comparisons of Ftt Schu S4 with *Fn* U112 are colored grey. Of the remaining probe sets, those representing genes which are intact in both strains are colored red, while the probe sets representing genes with one of the orthologs annotated as a pseudogene are colored blue.].

When transcription in the human virulent Ftt Schu S4 strain is compared to transcription in the human avirulent Fn U112 strain (Fig 4; S1 and S2 Tables), the largest group of shared genes falls into similar levels of expression (<5-fold). However, the majority of the genes that falls outside this range are transcribed at higher levels in Fn compared to Ftt. A number of these genes have low expression in Ftt either due to pseudogene formation or polar effects of pseudogenes and transposases. In contrast, most of the genes with high expression in Ftt are actually unique to Ftt and missing from Fn.

Of particular interest are shared genes that are more highly transcribed in Ftt when compared to Fn, as these may give insight into the higher virulence of Ftt for humans. Some of these include an acid phosphatase, an Emr secretion protein, an iron acquisition gene cluster (*fsl* operon), transposases (described above) and genes encoding hypothetical proteins (Table 1). EmrA proteins are typically the membrane fusion protein components (MFP) of tripartite efflux assemblies, which encompass multidrug efflux systems (RND) and Type I secretion systems (T1SS) [36, 37]. MFPs span the periplasm and connect the efflux transporter in the cytoplasmic membrane with a specific outermembrane porin to facilitate secretion across both membranes in one step [38]. MFPs are generally involved in transport of various substances (e.g. hemolysins, multidrug resistance). The OM porin TolC known to be utilized for MDR and T1S in other bacteria is involved in efflux of detergents, dyes and antibiotics in *Francisella*, and the transporter/MFP pair AcrAB also function in resistance to antibiotics; both contribute to the virulence of *Francisella* [39, 40]. Also, the EmrA1 MFP in Fth (LVS) has been shown to be required for resistance to oxidative stress and virulence [41]. The *emrA2* (FTT1654) gene in *Francisella* is flanked by two ORFs in an apparent operon: a predicted lipoprotein with a conserved domain of unknown function (FTT1653; DUF3568), and a putative inner membrane protein involved in fusaric acid resistance (FTT1655). All of these genes are highly transcribed in Ftt Schu S4 and in both of the *Fth* strains (OR960246 and LVS), but expressed at low levels in Fn U112.

The *fsl* gene cluster is likewise highly expressed in Ftt and both Fth strains, but transcribed at low levels in Fn. The *fsl* genes are required for siderophore synthesis in *Francisella* [42–44]. Siderophores facilitate iron uptake in bacteria by scavenging ferric iron in the surrounding environment and transporting it to the cytoplasm. The *fslA* gene product is a siderophore synthetase, and the other genes within the operon (*fslBCDEF*) are also required for siderophore synthesis/utilization [42, 44]. Mutations in these genes leads to poor growth in iron depleted medium [44], however there is another uptake system for ferrous iron (*feo*) that has been shown to compensate for the lack of siderophore synthesis *in vivo*, at least in Fth [45]. The high level of transcription of the ferric-siderophore operon in Ftt and *Fth* strains when compared to Fn during growth in CDM suggests that Ftt and Fth are experiencing iron limitation, but that Fn may have additional mean(s) to acquire iron under these conditions.

Acid phosphatases have been hypothesized to contribute to the virulence of *Francisella* by inhibition of the oxidative burst inside the phagosome [46]. There are 5–6 genes encoding acid phosphatases in the different *Francisella* species. It has been shown that four functional acid phosphatases AcpA, AcpB, AcpC and HapA contribute 90–99% of the acid phosphatase activity in Fn and Ftt and together are required for intramacrophage survival and virulence [47–49]. However it has also been reported that acid phosphatases do not contribute to *Ftt* virulence [50]. AcpA, which provides most of the acid phosphatase activity, is secreted *in vitro* [51], and translocated into the host macrophage cytosol by Fn and Ftt [52]. Higher level transcription of *acpA* in *Ftt* compared to *Fn* may influence the relative virulence these strains have for humans. Further investigation is necessary to definitively identify the role of AcpA in the virulence of different species/subspecies of *Francisella*.

FTT1140 (198 bp) and FTT1242 (1263 bp) are both annotated as encoding hypothetical proteins. FTT1140 is predicted to encode a short peptide of 65 aa, whereas the Fn ortholog (FTN1122) is predicted to be 129 aa. Interestingly, the Ftt protein appears to have a truncation at the N-terminus with respect to the Fn protein, and this region is predicted to contain a transmembrane segment (HMMTOP), suggesting that these proteins would have different subcellular locations within the two strains. FTT1242 contains a conserved domain (COG0172) found in seryl-tRNA synthetases. Interestingly, Fn contains two FTT1242 orthologs (FTN1260 and FTN1261) that share 65% identity; FTT1242 shares the closest identity to FTN1260 (95%) than to FTN1261 (61%). This suggests that the FTN1261 gene was deleted from Ftt/Fth during their evolutionary divergence from Fn. The relevance of these two hypothetical genes being transcribed at higher levels in Ftt is not clear.

### Comparison of transcription of Ftt and Fth

*Ftt* strain Schu S4 and *Fth* (wildtype) strain OR960246 are more closely related to each other than to Fn, however a number of differences in gene expression were observed when these strains were grown under identical conditions. Since Ftt is known to be more virulent in humans than Fth, some of these transcription differences may underlie their relative virulence (S3 and S4 Tables). Genes that are similar between these two strains but with significant transcription differences are noted in Tables 2 and 3.

The FTT1089-FTT1091 operon is highly expressed in Ftt in comparison to Fth. This operon encodes two isochorismatases (FTT1089 and FTT1091) and a transporter (FTT1090). Isochorismatases catalyze one of the steps in the conversion of isochorismate to 2-3-Dihydroxy Benzoic acid (2-3-DHBA), which itself can act as an iron chelator or can be diverted to form further complex siderophores [53]. It is unknown whether 2-3-DHBA is involved in iron uptake in *Francisella*. This operon appears intact in Ftt, whereas in *Fth* (both OR960246 and LVS), one of the isochorismatases (the ortholog of FTT1091) is a pseudogene due to insertion of a transposase, and in Fn, the transporter (the ortholog of FTT1090) is a pseudogene. In Fth the operon is not expressed, in contrast to Ftt Schu S4. Due to the differences in transcription and translation between *Ftt* and *Fth*, as well as the differences in siderophore (*fsl*) transcription mentioned above, this operon warrants further investigation.

Four different genes predicted to encode transporters were transcribed at higher levels in Ftt Schu S4 than in Fth OR960246: FTT0127, FTT0442, FTT0707, and FTT1004. Transposon insertions upstream of the FTT1004 and FTT1234 orthologs in Fth may explain their lower transcription, but it is unclear why the other genes are also expressed at lower levels in Fth. FTT1234 encodes a cholylglycine hydrolase that has been implicated in virulence in *Brucella* and *Listeria* [54, 55]. FTT0784, FTT1175, FTT1414, and FTT1784 encode hypothetical proteins whose orthologs are transcribed at lower levels in Fth; the presence of pseudogenes upstream in Fth possibly explains this differential expression. Orthologs to hypothetical genes FTT0185, FTT0845, and FTT1143, as well as a gene involved in valine biosynthesis, FTT0642, were transcribed at higher levels in Fth OR960246 compared to Ftt Schu S4 (Table 3).

There are interesting differences in the presence and expression of genes predicted to be involved in polyamine synthesis between the various Ft strains (S1 and S3 Tables). The response of bacterial pathogens to polyamines has been associated with various aspects of virulence. It has been shown that the Fth and Ftt response to exogenous spermine and spermidine alters global gene expression patterns, including an upregulation of transcription from IS*Ftu1* and IS*Ftu2* insertion elements, which also increases expression of genes adjacent to these elements [35]. Growth of Ft in spermine or spermidine downregulates expression of TNFα and IL-12 by infected macrophages. CDM contains spermine, and was utilized in the studies presented here.

Putrescine and spermidine are the major endogenous polyamines in bacteria, and they can synthesize putrescine either directly from ornithine or indirectly via arginine, and then convert putrescine to spermidine; bacteria do not synthesize spermine. Ftt Schu S4 contains a cluster of genes, *speD* (S-adenosylmethionine carboxylase), *speE* (spermidine synthase), *speA* (arginine decarboxylase), FTT0434 (agmatine deiminase; *aguA*) and FTT0435 (N-carbamoylputrescine amidohydrolase; *aguB*) in an apparent operon. In *Fth* strains, *speD* and *speE* are intact while *speA*, *aguA* and *aguB* are pseudogenes, and the entire region is absent in *Fn* U112. Thus Fth strains might be predicted to not be able to convert arginine to putrescine because they lack the three genes necessary, but to maintain the ability to convert putrescine to spermidine due to the presence of *speD* and *speE*, whereas Fn lacks this entire pathway. Alternatively, bacteria can convert ornithine directly to putrescine by ornithine decarboxylase (*speC*), but no *speC* ortholog has been annotated in the Ft genome. The gene annotated as diaminopimelate decarboxylase (*lysA;* FTT0027) may fulfill this function as SpeC. As mentioned above, this gene lies within the siderophore synthesis operon and is also annotated as *fslC* for its role in iron uptake. The Ft siderophore resembles rhizoferrin and has a putrescine backbone [42], and FTT0027 is a member of the pyridoxal-dependent decarboxylases (pfam02784) which include ornithine decarboxylases. Thus this gene may provide synthesis of putrescine directly from ornithine.

Interestingly, even though *speD* and *speE* are intact in the *Fth* strains, their expression is significantly lower than their orthologs in Ftt Schu S4 when grown under identical conditions. The lower expression of *speD* and *speE*, and absence of *speA*, *aguA* and *aguB* in Fth compared to Ftt may contribute to the relative lower virulence of this subspecies in humans. Considering that Fn lacks all five genes and exhibits the lowest virulence of all three subspecies in humans, this suggests that endogenous polyamine synthesis may contribute to Ft virulence. Endogenous polyamines influence many aspects of bacteria, including transcription, translation, cell growth, and stress resistance, and polyamines have been shown to modulate the virulence of a variety of pathogens, including *Shigella*, *Yersinia*, and *Salmonella* [56].

Additional genes more highly expressed in Ftt compared to Fth may contribute to enhanced virulence (S3 Table). For example, NADPH-quinone reductase (*mdaB*) catalyzes the reduction of quinones and may help protect against damage by free radicals and reactive oxygen species within the macrophage more effectively. The *tlyC* gene, which encodes a protein with cystathionine beta-synthase (CBS) domains found in transporters, is more highly expressed in Ftt, but it is not clear whether this protein has any hemolytic activity despite being annotated as a hemolysin [57]. The *pdpD* gene within the *Francisella* pathogenicity island is expressed higher in Ftt than in Fth. Differences in this gene distinguish the different *Francisella* subspecies, with Fn expressing a PdpD protein that is 50 aa larger than that in Ftt. In contrast, the *pdpD* gene is essentially missing from Fth, with only a small portion of the C-terminal coding sequence remaining [10]. The shared probes within the microarray were designed to this portion, and transcription was reduced 12-fold in Fth vs Ftt, while the transcription of the downstream genes (*iglABCD*) was not altered. PdpD contributes to the virulence of Fn in chicken embryos and mice, but it is not required for intracellular growth *in vitro* [58]. *Francisella* spp have Type IV pilus genes that are involved in secretion and pilus fiber expression [59]. The high transcription of *pilT* and *pilA* in Ftt Schu S4 compared to Fth may contribute to its higher virulence.

### Comparison of transcription of Fth OR960246 and Fth LVS

**Fig 5:**
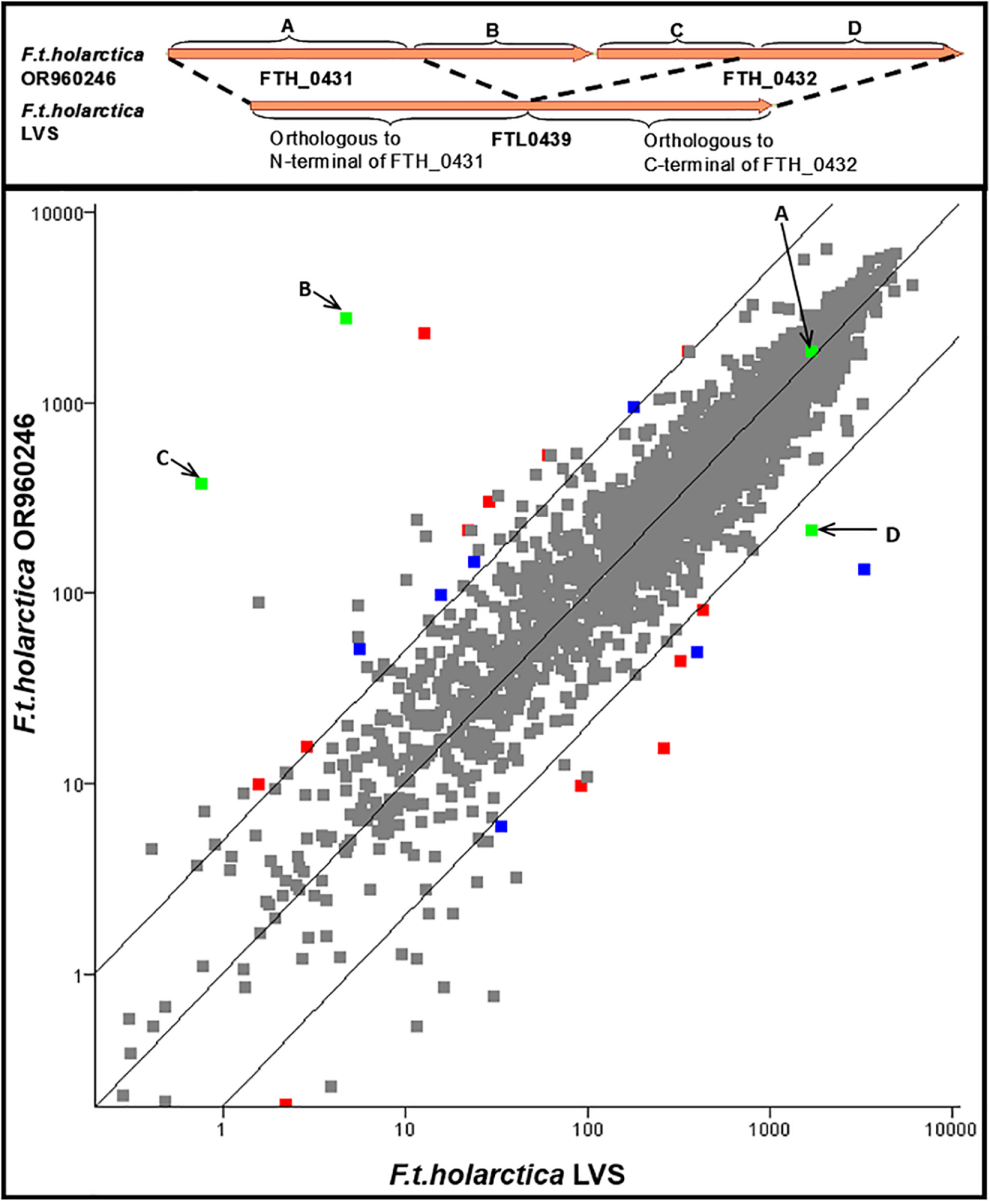
Comparison of Transcriptomes of Strains of *Fth* OR960246 *and Fth* LVS. The genomic region of *Fth* LVS FTL0439 compared to the orthologous region FTH0431 and FTH0432 in *Fth* OR960246 is shown in the top panel. The bottom panel shows the comparison of gene expression in *Fth* OR960246 and *Fth* LVS strains. Each point represents a probe set monitoring the transcription of a unique computationally predicted gene (average of three biological replicates). Probe sets that have differential expression of less than five fold and/or a p-value greater than 0.05 in one way comparisons of Fth OR960246 with *Fth* LVS are colored grey. Of the remaining probe sets, those representing genes which are intact in both strains are colored red, while the probe sets representing genes with one of the orthologs annotated as a pseudogene are colored blue. The probe sets covering the FTL0439/FTH0431-0432 region are colored green and indicated by letter corresponding to top panel.

The Fth LVS strain is highly attenuated for virulence and was derived by repeated passage in the laboratory. The LVS genome differs from that of the wildtype Fth OR960246 strain by a number of SNPs and indels. Not surprisingly, comparative transcriptional analysis revealed that the two *Fth* strains have very similar expression profiles, mirroring their highly similar genome architecture (Fig 5, Tables 4 and 5). There are two paralogs, FTH_0431 (*fopC*) and FTH_0432, in Fth that share 50.4% homology and that have undergone recombination to form a fusion gene in LVS, FTL_0439. The difference in architecture of the gene between the two Fth strains is shown in Fig 5. In Fth OR960246 and in Ftt Schu4, the two genes in the orthologous genomic region have different levels of expression; FTH_0431 (*fopC*) is highly expressed and FTH_0432 has a much lower expression. Due to the fusion of the C-terminal region of FTH_0432 (probe set region D) to the N-terminal region of FTH_0431 (probe set region A) in LVS, the two regions (probe set regions A and D) have the same level of expression.

**Table 4:** Genes with High Expression in *Fth* OR960246 Compared to *Fth* LVS.

Locus in Fth OR960246	Locus in Fth LVS	Fold Difference*	Product
FTH_0160	FTL_0167	10	DNA helicase
FTH_0384	None	182	pilA
FTH_0431	None	494	fopC
FTH_0432	None	583	hypothetical protein
FTH_0752	FTL_0750	5	hypothetical protein

* ≥ 5 fold

p ≤ 0.05

**Table 5:** Genes with Low Expression in *Fth* OR960246 Compared to *Fth* LVS.

Locus in Fth OR960246	Locus in Fth LVS	Fold Difference*	Product
FTH_0061	None	6	predicted pseudogene
FTH_0383	None	25	predicted pseudogene
FTH_1250	FTL_1277	5	nagC
FTH_1328	FTL_1363	7	Esterase

*≥ 5 fold

p ≤ 0.05

FopC (FupC) is a paralog of *figE* (*fslE*) of the *fig* operon described in the sections above. It has been implicated in the high affinity uptake of ferrous iron [60]. The ortholog of FTH_0431 in *Ftt* Schu S4 (FTT_0918) was shown to be required for virulence and a strain with a mutation in this gene was effective as a live vaccine [61]. Reintroduction of the FTH_0431 (*fopC*) along with *pilA* (FTH_0384) into LVS restored virulence to a level indistinguishable from the wild type *Fth* strains [29]. FopC has also been shown to help in avoiding IFN gamma-mediated immune defenses [62]. No studies have been performed to understand the biological function and immunogenicity of the downstream FTH_0432. It is probable that one of the major factors responsible for the attenuation of LVS is the lack of the C-terminal region of FTH0431.

In addition to FTL_0431, the other gene that is highly expressed in OR960246 when compared to LVS is a *pilA* gene. Pilus genes have been shown previously to be required for virulence [63]. The genes that are highly expressed in LVS and expressed at lower levels in OR960246 are an esterase and *nagC*. NagC is involved in the synthesis and catabolism of glucosaminoglycans and chitin [64]. The biological role of these genes and their effect on virulence is not yet known.

### Limitations

This study represents a comparison of the transcriptome of multiple strains of *Francisella* grown under identical conditions in the chemically-defined Chamberlain’s medium. We have noted the genes with 5-fold or greater differences in transcription (p < 0.05) between the strains, and suggested that some of these differences may contribute to the relative virulence of these strains. However this technique was utilized as a discovery tool, and further analyses like 1) RT-PCR confirmation 2) comparative proteomic analyses 3) growth analyses in different media and cells, and 4) animal infection studies will be required to validate these expression differences as the bases of differences in virulence. Additionally, different growth conditions (e.g. medium, temperature, etc) are likely to highlight additional differences in gene expression between species/subspecies. There are also multiple genes whose expression differed from 2- to 5-fold between strains (p <0.05) that were not discussed but that could also be of significance. Of course numerous other factors not addressed here may also contribute to relative expression of the corresponding proteins, such as message half-life, translation efficiency, and post translational modifications.

## Summary and Future Directions

The *Francisella* shared probe set microarray designed for this study allowed us to perform comparative transcription studies of Ftt, Fth, and Fn strains grown under identical conditions. In the past, microarray studies have been performed with probes designed and optimized for Ftt, but utilized for additional *Francisella* strains [43, 65]. These approaches were useful, but had significant limitations due to the variability of genome sequences across the *Francisella* subspecies. However, due to high similarity in the genomes of these subspecies, we were able to design a consensus microarray for Ftt, Fth, and Fn that represented the core set of shared genes, as well as the species-specific genes and the inter-genic regions. Our studies allowed us to perform direct comparisons of relative transcript levels across species/subspecies, and highlighted potentially interesting differences in gene expression that may be the basis for further studies to identify the underlying cause of the relative virulence of these strains in humans.

## Supporting Information

S1 Table Genes with High Expression (≥ 5 fold and p≤ 0.05) in *Ftt* Compared to *Fn*. (DOCX)

S2 Table Genes with High Expression (≥ 5 fold and p≤ 0.05) in Fn compared to Ftt. (DOCX)

S3 Table ORFs with high expression (≥ 5 fold and p≤ 0.05) in Ftt compared to Fth. (DOCX)

S4 Table ORFs with high expression (≥ 5 fold and p≤ 0.05) in Fth compared to Ftt. (DOCX)

S5 Table Genes with High Expression (≥ 5 fold and p≤ 0.05) in *Fth* Compared to *Fn*. (DOCX)

S6 Table Genes with High Expression (≥ 5 fold and p≤ 0.05) in *Fn* Compared to *Fth*. (DOCX)

S7 Table Expression of Transposases. (DOCX)
