# Supplementary material for: Comparative Transcriptional Analyses of Francisella tularensis and Francisella novicida
Source: PLoS One. 2016 Aug 18;11(8):e0158631. doi: 10.1371/journal.pone.0158631 (PMC4990168; doi:10.1371/journal.pone.0158631)
Supplement: S1 Table — (DOCX) [file pone.0158631.s001.docx]

**Supporting Information:**

S1 Table: Genes with High Expression (≥ 5 fold and p≤ 0.05) in *Ftt* Compared to *Fn.*

|  |  |  |  |  |  |  |
| --- | --- | --- | --- | --- | --- | --- |
| **Locus in Schu S4** | **Locus in U112** | **Intensity in Schu S4** | **Intensity in U112** | **Fold Difference** | **Gene in**  **Schu S4** | **Product** |
|  |  |  |  |  |  |  |
|  |  |  |  |  |  |  |
| **Gene is intact in Schu S4 and its ortholog in U112 is also intact** | | | | | | |
|  | | | | | | |
| FTT0027 | FTN_1684 | 110.71 | 6.76 | 16 | lysA1 | pyridoxal-dependent decarboxylase |
| FTT0028 | FTN_1683 | 89.73 | 16.25 | 6 | FTT0028 | drug:H+ antiporter-1 (DHA1) family protein |
| FTT0029 | FTN_1682 | 172.12 | 10.37 | 17 | frgA (fslA) | siderophore biosynthesis protein |
| FTT0103 | FTN_1612 | 95.19 | 18.5 | 5 | FTT0103 | hypothetical protein |
| FTT0221 | FTN_0090 | 1626.2 | 304.99 | 5 | acpA | acid phosphatase (precursor) |
| FTT1140 | FTN_1122 | 3118.51 | 336.84 | 9 | FTT1140 | hypothetical protein |
| FTT1242 | FTN_1260 | 3253.89 | 323.45 | 10 | FTT1242 | hypothetical protein |
| FTT1653 | FTN_0030 | 2297.67 | 23.25 | 99 | FTT1653 | hypothetical protein |
| FTT1654 | FTN_0029 | 1493.31 | 7.85 | 190 | emrA2 | HlyD family secretion protein |
| FTT1655 | FTN_0028 | 997.17 | 13.92 | 72 | FTT1655 | hypothetical protein |
|  |  |  |  |  |  |  |
| **Gene is intact in Schu S4 and its ortholog in U112 is a pseudogene** | | | | | | |
|  | | | | | | |
| FTT1450 | FTN_1419 | 1323.83 | 9.92 | 133 | wbtM | dTDP-D-glucose 4,6-dehydratase |
|  |  |  |  |  |  |  |
| **Gene is intact in Schu S4 and its ortholog in U112 is absent** | | | | | | |
|  | | | | | | |
| FTT0016 | None | 1589.76 | 1 | 1590 | FTT0016 | hypothetical protein |
| FTT0300 | None | 673.94 | 2.25 | 300 | FTT0300 | hypothetical protein |
| FTT0301 | None | 217.24 | 1 | 217 | FTT0301 | hypothetical protein |
| FTT0395 | None | 528.38 | 11.54 | 46 | FTT0395 | hypothetical protein |
| FTT0430 | None | 830.96 | 22.57 | 37 | speH | S-adenosylmethionine decarboxylase |
| FTT0431 | None | 418.66 | 7.45 | 56 | speE | spermidine synthase |
| FTT0432 | None | 557.88 | 1 | 558 | speA | putative arginine decarboxylase |
| FTT0433 | None | 468.94 | 1.5 | 312 | FTT0433 | putative arginine decarboxylase |
| FTT0434 | None | 361.46 | 1.49 | 243 | FTT0434 | hypothetical protein |
| FTT0435 | None | 337.77 | 1.8 | 188 | FTT0435 | Carbon-nitrogen hydrolase family protein |
| FTT0496 | None | 312.33 | 3.57 | 87 | FTT0496 | hypothetical protein |
| FTT0520 | None | 158.14 | 9.91 | 16 | FTT0520 | hypothetical protein |
| FTT0525 | None | 174.64 | 1.14 | 153 | FTT0525 | hypothetical protein |
| FTT0572 | None | 240.78 | 6.04 | 40 | FTT0572 | POT family protein |
| FTT0573 | None | 202.21 | 1.45 | 139 | alr | alanine racemase |
| FTT0602 | None | 290.87 | 4.01 | 72 | FTT0602 | hypothetical protein |
| FTT0677 | None | 168.49 | 1 | 168 | FTT0677 | hypothetical protein |
|  |  |  |  |  |  |  |

(Continued)

**S1 Table (continued).**

|  |  |  |  |  |  |  |
| --- | --- | --- | --- | --- | --- | --- |
| **Locus in Schu S4** | **Locus in U112** | **Intensity in Schu S4** | **Intensity in U112** | **Fold Difference** | **Gene in**  **Schu S4** | **Product** |
|  |  |  |  |  |  |  |
|  |  |  |  |  |  |  |
| FTT0727 | None | 241.55 | 2.84 | 85 | FTT0727 | hypothetical protein |
| FTT0742 | None | 126.35 | 12.92 | 10 | FTT0742 | hypothetical protein |
| FTT0748 | None | 1686.91 | 5.3 | 318 | FTT0748 | hypothetical protein |
| FTT0794 | None | 992.45 | 3.38 | 294 | FTT0794 | hypothetical protein |
| FTT0795 | None | 1322.55 | 2.68 | 494 | FTT0795 | hypothetical protein |
| FTT0796 | None | 1228.84 | 1.18 | 1041 | FTT0796 | hypothetical protein |
| FTT0890 | None | 2242.63 | 32.36 | 69 | FTT0890 | type IV pili fiber protein |
| FTT0939 | None | 543.67 | 2.38 | 228 | add1 | None |
| FTT0958 | None | 382.12 | 2.73 | 140 | FTT0958 | Short-chain dehdyrogenase |
| FTT1068 | None | 263.32 | 1 | 263 | FTT1068 | None |
| FTT1069 | None | 183.92 | 9.38 | 20 | FTT1069 | None |
| FTT1072 | None | 334.14 | 1.19 | 282 | FTT1072 | hypothetical protein |
| FTT1077 | None | 103.05 | 1.37 | 75 | FTT1077 | hypothetical protein |
| FTT1078 | None | 331.14 | 5.76 | 57 | FTT1078 | hypothetical protein |
| FTT1079 | None | 242.94 | 3.29 | 74 | FTT1079 | hypothetical protein |
| FTT1080 | None | 149.36 | 1 | 149 | FTT1080 | hypothetical protein |
| FTT1081 | None | 195.38 | 1.37 | 143 | FTT1081 | hypothetical protein |
| FTT1122 | None | 786.03 | 3.53 | 223 | FTT1122 | hypothetical lipoprotein |
| FTT1174 | None | 117.46 | 3.61 | 33 | FTT1174 | hypothetical protein |
| FTT1175 | None | 142.58 | 1.32 | 108 | FTT1175 | hypothetical membrane protein |
| FTT1263 | None | 4094.63 | 1.76 | 2330 | FTT1263 | hypothetical protein |
| FTT1308 | None | 344.75 | 12.36 | 28 | FTT1308 | None |
| FTT1395 | None | 482.65 | 2.42 | 199 | FTT1395 | ATP-dependent DNA helicase |
| FTT1450 | None | 1178.43 | 16.27 | 72 | wbtM | dTDP-D-glucose 4,6-dehydratase |
| FTT1451 | None | 1615.45 | 10.05 | 161 | wbtL | Glucose-1-phosphate thymidylyltransferase |
| FTT1452 | None | 1922.18 | 2.78 | 693 | wbtK | Glycosyltransferase |
| FTT1453 | None | 1052.88 | 1.36 | 774 | wzx | O-antigen flippase |
| FTT1454 | None | 2331.4 | 1.37 | 1697 | wbtJ | formyl transferase |
| FTT1455 | None | 2379.49 | 2.06 | 1156 | wbtI | sugar transamine/perosamine synthetase |
| FTT1458 | None | 2267.22 | 1 | 2267 | wzy | Membrane protein/O-antigen protein |
| FTT1462 | None | 1933.6 | 27.57 | 70 | wbtC | UDP-glucose 4-epimerase |
| FTT1463 | None | 2466.71 | 2.42 | 1018 | wbtB | galactosyl transferase |
| FTT1580 | None | 710.46 | 1.26 | 563 | FTT1580 | None |
| FTT1581 | None | 603.37 | 2.79 | 216 | FTT1581 | Endonuclease |
| FTT1594 | None | 471.46 | 16.34 | 29 | FTT1594 | transcriptional regulator |
| FTT1614 | None | 177.22 | 7.79 | 23 | FTT1614 | hypothetical protein |
| FTT1659 | None | 704.4 | 8.96 | 79 | FTT1659 | hypothetical protein |
| FTT1666 | None | 763.02 | 3.11 | 246 | FTT1666 | 3-hydroxyisobutyrate dehydrogenase |
| FTT1787 | None | 364.83 | 1.36 | 267 | FTT1787 | Transporter, LysE family |
| FTT1789 | None | 148.59 | 6.26 | 24 | FTT1789 | hypothetical protein |
| FTT1791 | None | 322.52 | 1 | 323 | FTT1791 | hypothetical protein |
|  |  |  |  |  |  |  |

(Continued)

**S1 Table (continued).**

|  |  |  |  |  |  |  | | |
| --- | --- | --- | --- | --- | --- | --- | --- | --- |
| **Locus in Schu S4** | **Locus in**  **U112** | **Intensity in Schu S4** | **Intensity in U112** | **Fold Difference** | **Gene in**  **Schu S4** | **Product** | | |
|  |  |  |  |  |  |  | | |
|  |  |  |  |  |  |  | | |
| **Gene is a pseudogene in Schu S4 and its ortholog in U112 is absent** | | | | | | |  |  |
|  | | | | | | |  |  |
| FTT0010 | None | 161.92 | 1.24 | 131 | FTT0010 | None | | |
| FTT0011 | None | 122.64 | 14.07 | 9 | FTT0011 | None | | |
| FTT0521 | None | 104.36 | 1 | 104 | FTT0521 | None | | |
| FTT0600 | None | 1070.75 | 14.89 | 72 | FTT0600 | None | | |
| FTT0921 | None | 119.79 | 5.51 | 22 | FTT0921 | None | | |
| FTT1070 | None | 269.07 | 3.09 | 87 | FTT1070 | None | | |
| FTT1519 | None | 2237.54 | 1.25 | 1787 | FTT1519 | None | | |
| FTT1719 | None | 601.44 | 2.67 | 225 | FTT1719 | None | | |
| FTT1788 | None | 327.53 | 1.09 | 300 | iciA | None | | |
| FTT1790 | None | 118.99 | 4.02 | 30 | FTT1790 | None | | |
|  |  |  |  |  |  |  | | |
| **Gene is a pseudogene in Schu S4 and its ortholog in U112 is also a pseudogene** | | | | | | |  |  |
|  | | | | | | |  |  |
| FTT1717 | FTN_1327 | 111.57 | 10.82 | 10 | FTT1717 | None | | |
|  |  |  |  |  |  |  | | |
