# Supplementary material for: Comparative Transcriptional Analyses of Francisella tularensis and Francisella novicida
Source: PLoS One. 2016 Aug 18;11(8):e0158631. doi: 10.1371/journal.pone.0158631 (PMC4990168; doi:10.1371/journal.pone.0158631)
Supplement: S2 Table — (DOCX) [file pone.0158631.s002.docx]

S2 Table: Genes with High Expression (≥ 5 fold and p≤ 0.05) in Fn compared to Ftt*.*

|  |  |  |  |  |  |  |
| --- | --- | --- | --- | --- | --- | --- |
| **Locus in U112** | **Locus in**  **Schu S4** | **Intensity in Schu S4** | **Intensity in U112** | **Fold Difference** | **Gene in U112** | **Product** |
|  |  |  |  |  |  |  |
|  |  |  |  |  |  |  |
| **Gene is intact in U112 and its ortholog in Schu S4 is also intact** | | | | | | |
|  | | | | | | |
| FTN_0032 | FTT1651 | 138.35 | 807.55 | 6 | FTN_0032 | hypothetical protein |
| FTN_0057 | FTT1645 | 59.12 | 306.54 | 5 | FTN_0057 | major facilitator superfamily (MFS) transport protein |
| FTN_0058 | FTT1644 | 43.06 | 339.41 | 8 | FTN_0058 | beta-fructofuranosidase |
| FTN_0067 | FTT0248 | 60.4 | 305.75 | 5 | FTN_0067 | hypothetical protein |
| FTN_0081 | FTT0241 | 31.76 | 613.74 | 19 | FTN_0081 | hypothetical protein |
| FTN_0089 | FTT0222 | 94.83 | 502.47 | 5 | FTN_0089 | allophanate hydrolase subunit 2 |
| FTN_0092 | FTT0219 | 124.06 | 774.83 | 6 | FTN_0092 | inorganic phosphate transporter (PiT) family protein |
| FTN_0100 | FTT1685 | 69.33 | 560.39 | 8 | FTN_0100 | hypothetical protein |
| FTN_0128 | FTT0006 | 54.83 | 466.41 | 9 | FTN_0128 | metabolite:H+ symporter (MHS) family protein |
| FTN_0150 | FTT0174 | 149.62 | 885.45 | 6 | FTN_0150 | YggT family protein |
| FTN_0360 | FTT0845 | 6.94 | 326.33 | 47 | FTN_0360 | hypothetical protein |
| FTN_0361 | FTT0846 | 26.48 | 229.65 | 9 | FTN_0361 | deoxyribodipyrimidine photolyase |
| FTN_0362 | FTT0847 | 6.05 | 170.32 | 28 | FTN_0362 | deoxyribodipyrimidine photolyase-related protein |
| FTN_0471 | FTT1289 | 45.89 | 1844.26 | 40 | FTN_0471 | NADPH-dependent FMN reductase |
| FTN_0498 | FTT0401 | 25.86 | 134.62 | 5 | FTN_0498 | hypothetical protein |
| FTN_0527 | FTT0428 | 71.57 | 440.09 | 6 | thrC | threonine synthase |
| FTN_0590 | FTT0499 | 51.11 | 270.86 | 5 | FTN_0590 | hypothetical protein |
| FTN_0590 | FTT0500 | 28.27 | 212.53 | 8 | FTN_0590 | hypothetical protein |
| FTN_0596 | FTT0506 | 11.42 | 122.39 | 11 | FTN_0596 | hypothetical protein |
| FTN_0717 | FTT0744 | 27.17 | 149.07 | 5 | FTN_0717 | hypothetical protein |
| FTN_0717 | FTT0745 | 36.94 | 418.79 | 11 | FTN_0717 | hypothetical protein |
| FTN_0740 | FTT0566 | 24.46 | 480.72 | 20 | FTN_0740 | hypothetical protein |
| FTN_0855 | FTT0975 | 342.98 | 2381.49 | 7 | FTN_0855 | hypothetical protein |
| FTN_0856 | FTT0976 | 398.1 | 2197.79 | 6 | FTN_0856 | thioredoxin |
| FTN_0878 | FTT0998 | 98.73 | 1436.75 | 15 | FTN_0878 | hypothetical protein |
| FTN_0886 | FTT1006 | 15.58 | 227.32 | 15 | FTN_0886 | sugar:cation symporter family protein |
| FTN_0962 | FTT0553 | 281.13 | 1885.84 | 7 | FTN_0962 | hypothetical protein |
| FTN_0963 | FTT0552 | 156.1 | 1263.96 | 8 | FTN_0963 | NAD-dependent aldehyde dehydrogenase |
| FTN_0972 | FTT0543 | 19.4 | 181.21 | 9 | FTN_0972 | hypothetical protein |
| FTN_1032 | FTT0651 | 96.7 | 781.5 | 8 | FTN_1032 | proton-dependent oligopeptide transporter |
|  |  |  |  |  |  |  |

(Continued)

**S2 Table (continued).**

|  |  |  |  |  |  |  |
| --- | --- | --- | --- | --- | --- | --- |
| **Locus in U112** | **Locus in**  **Schu S4** | **Intensity in Schu S4** | **Intensity in U112** | **Fold Difference** | **Gene in U112** | **Product** |
|  |  |  |  |  |  |  |
|  |  |  |  |  |  |  |
| FTN_1041 | FTT0642 | 192.4 | 3436.78 | 18 | ilvN | acetolactate synthase small subunit |
| FTN_1043 | FTT0640 | 116.95 | 1224.81 | 10 | ilvD | dihydroxy-acid dehydratase |
| FTN_1044 | FTT0638 | 105.74 | 1794.71 | 17 | FTN_1044 | hypothetical protein |
| FTN_1124 | FTT1143 | 33.77 | 374.22 | 11 | FTN_1124 | hypothetical protein |
| FTN_1169 | FTT1191 | 3.38 | 327.87 | 97 | FTN_1169 | peptidase, M20 family |
| FTN_1170 | FTT1193 | 119.04 | 1266.6 | 11 | FTN_1170 | hypothetical protein |
| FTN_1171 | FTT1194 | 151.73 | 1391.56 | 9 | FTN_1171 | hypothetical protein |
| FTN_1386 | FTT1420 | 41.67 | 1214.82 | 29 | FTN_1386 | hypothetical protein |
| FTN_1398 | FTT1428 | 43.39 | 361.14 | 8 | FTN_1398 | acetyltransferase |
| FTN_1413 | FTT1443 | 30.21 | 423.6 | 14 | FTN_1413 | AAA family-ATPase |
| FTN_1457 | FTT1549 | 26.84 | 744.01 | 28 | FTN_1457 | hypothetical protein |
| FTN_1458 | FTT1550 | 65.98 | 360.64 | 5 | FTN_1458 | hypothetical protein |
| FTN_1459 | FTT1551 | 23.85 | 493.43 | 21 | FTN_1459 | short chain dehydrogenase |
| FTN_1529 | FTT1520 | 26.25 | 341.14 | 13 | FTN_1529 | APC family protein |
| FTN_1554 | FTT0158 | 13.64 | 114.26 | 8 | FTN_1554 | hypothetical protein |
| FTN_1589 | FTT0126 | 19.3 | 1017.2 | 53 | oppF | PepT family protein |
| FTN_1590 | FTT0125 | 36.27 | 1099.13 | 30 | oppD | PepT family protein |
| FTN_1618 | FTT0093 | 102.49 | 632.91 | 6 | FTN_1618 | hypothetical protein |
| FTN_1620 | FTT0091 | 62.21 | 2316.32 | 37 | appB | cytochrome bd-II terminal oxidase subunit II |
| FTN_1696 | FTT0013 | 59.08 | 299.99 | 5 | FTN_1696 | hypothetical protein |
| FTN_1716 | FTT1737 | 89.94 | 1009.25 | 11 | kdpC | potassium-transporting ATPase C |
| FTN_1717 | FTT1738 | 121.62 | 1146.64 | 9 | kdpB | potassium-transporting ATPase B |
|  |  |  |  |  |  |  |
| **Gene is intact in U112 and its ortholog in Schu S4 is a pseudogene** | | | | | | |
|  | | | | | | |
| FTN_0003 | FTT0003 | 52.27 | 513.87 | 10 | FTN_0003 | metabolite:H+ symporter (MHS) family protein |
| FTN_0022 | FTT1662 | 17.83 | 262.87 | 15 | FTN_0022 | histidine acid phosphatase |
| FTN_0086 | FTT0225 | 62.14 | 860.19 | 14 | FTN_0086 | metabolite:H+ symporter (MHS) family protein |
| FTN_0093 | FTT0218 | 42.15 | 454.65 | 11 | FTN_0093 | cytochrome b561 family protein |
| FTN_0094 | FTT0217 | 32.43 | 187.51 | 6 | FTN_0094 | transcriptional regulator, LysR family |
| FTN_0103 | FTT1682 | 12.94 | 428.14 | 33 | FTN_0103 | hypothetical protein |
| FTN_0115 | FTT1743 | 11.68 | 123.58 | 11 | FTN_0115 | Na+/H+ antiporter |
| FTN_0127 | FTT0005 | 23.73 | 499.65 | 21 | gabD | succinate semialdehyde dehydrogenase (NAD(P)+ dependent) |
|  |  |  |  |  |  |  |

(Continued)

**S2 Table (continued).**

|  |  |  |  |  |  |  |
| --- | --- | --- | --- | --- | --- | --- |
| **Locus in U112** | **Locus in**  **Schu S4** | **Intensity in Schu S4** | **Intensity in U112** | **Fold Difference** | **Gene in U112** | **Product** |
|  |  |  |  |  |  |  |
|  |  |  |  |  |  |  |
| FTN_0148 | FTT0172 | 36.12 | 686.35 | 19 | FTN_0148 | hypothetical protein |
| FTN_0176 | FTT0201 | 7.53 | 296.59 | 39 | FTN_0176 | serine permease |
| FTN_0287 | FTT1641 | 8.78 | 127.98 | 15 | FTN_0287 | type I restriction-modification system, subunit R (restriction) |
| FTN_0313 | FTT1619 | 31.73 | 185.31 | 6 | FTN_0313 | acetyltransferase |
| FTN_0359 | FTT0844 | 26.05 | 583.2 | 22 | FTN_0359 | dehydrogenase |
| FTN_0409 | FTT0883 | 100.8 | 630.19 | 6 | adhC | alcohol dehydrogenase |
| FTN_0525 | FTT0426 | 59.97 | 833.23 | 14 | thrA | aspartate kinase I |
| FTN_0526 | FTT0427 | 128.29 | 713.24 | 6 | thrB | homoserine kinase |
| FTN_0588 | FTT0497 | 72.8 | 773.77 | 11 | FTN_0588 | asparaginase |
| FTN_0635 | FTT0724 | 64.67 | 621.2 | 10 | FTN_0635 | serine-type D-Ala-D-Ala carboxypeptidase |
| FTN_0776 | FTT1098 | 49.16 | 339.5 | 7 | FTN_0776 | DNA and RNA helicases Superfamily I protein |
| FTN_0807 | FTT0929 | 59.31 | 888.3 | 15 | FTN_0807 | short chain dehydrogenase |
| FTN_0808 | FTT0930 | 91.06 | 791.36 | 9 | FTN_0808 | acetoacetate decarboxylase |
| FTN_0811 | FTT0933 | 22.53 | 344.76 | 15 | birA | biotin--acetyl-CoA-carboxylase ligase |
| FTN_0854 | FTT0974 | 363.83 | 2283.16 | 6 | FTN_0854 | hypothetical protein |
| FTN_0876 | FTT0996 | 13.48 | 191.17 | 14 | FTN_0876 | cardiolipin synthase |
| FTN_0973 | FTT0542 | 99.11 | 2261.07 | 23 | FTN_0973 | peroxiredoxin |
| FTN_1040 | FTT0643 | 50.52 | 2627.39 | 52 | ilvC | ketol-acid reductoisomerase |
| FTN_1042 | FTT0641 | 201.17 | 2353.15 | 12 | ilvB | acetolactate synthase |
| FTN_1044 | FTT0637 | 132.08 | 920.31 | 7 | FTN_1044 | hypothetical protein |
| FTN_1117 | FTT1135 | 56.9 | 592.9 | 10 | FTN_1117 | ATP binding protein |
| FTN_1121 | FTT1139 | 57.07 | 287.36 | 5 | phrB | deoxyribodipyrimidine photolyase |
| FTN_1125 | FTT1144 | 32.05 | 193.08 | 6 | FTN_1125 | short chain dehydrogenase |
| FTN_1143 | FTT1162 | 59.82 | 1661.77 | 28 | FTN_1143 | 4Fe-4S ferredoxin |
| FTN_1170 | FTT1192 | 76.04 | 1259.91 | 17 | FTN_1170 | hypothetical protein |
| FTN_1172 | FTT1195 | 116.15 | 1082.41 | 9 | FTN_1172 | hypothetical protein |
| FTN_1186 | FTT1209 | 65.55 | 3418.98 | 52 | pepO | M13 family metallopeptidase |
| FTN_1280 | FTT1261 | 35.71 | 596.88 | 17 | FTN_1280 | tryptophan repressor binding protein-like flavidoxin |
| FTN_1399 | FTT1429 | 49.93 | 411.16 | 8 | FTN_1399 | hypothetical protein |
| FTN_1454 | FTT1545 | 55.29 | 410 | 7 | FTN_1454 | NAD/FAD-binding protein |
| FTN_1455 | FTT1546 | 35.9 | 305.94 | 9 | FTN_1455 | hypothetical protein |
| FTN_1456 | FTT1547 | 43.23 | 888.72 | 21 | cfa | cyclopropane fatty acid synthase, methyltransferase |
| FTN_1466 | FTT1558 | 61.17 | 518.71 | 8 | FTN_1466 | hypothetical protein |
| FTN_1474 | FTT1565 | 227.86 | 2372.31 | 10 | bglX | glycosyl hydrolase family 3 |
| FTN_1530 | FTT1521 | 31.56 | 266.37 | 8 | lysA | diaminopimelate decarboxylase |
| FTN_1533 | FTT0358 | 21.7 | 322.07 | 15 | FTN_1533 | hypothetical protein |
| FTN_1543 | FTT0170 | 22.17 | 286.66 | 13 | FTN_1543 | hypothetical protein |
|  |  |  |  |  |  |  |

(Continued)

**S2 Table (continued).**

|  | |  |  |  |  |  |  |
| --- | --- | --- | --- | --- | --- | --- | --- |
| **Locus in U112** | | **Locus in**  **Schu S4** | **Intensity in Schu S4** | **Intensity in U112** | **Fold Difference** | **Gene in U112** | **Product** |
|  | |  |  |  |  |  |  |
|  | |  |  |  |  |  |  |
| FTN_1591 | FTT0124 | | 31.84 | 849.97 | 27 | oppC | PepT family protein |
| FTN_1592 | | FTT0123 | 19.66 | 1053.34 | 54 | oppB | peptide/opine/nickel uptake transporter (PepT) family protein |
| FTN_1593 | | FTT0122 | 129.36 | 901.23 | 7 | oppA | ABC-type oligopeptide transport system, periplasmic component |
| FTN_1619 | | FTT0092 | 41.99 | 1866.05 | 44 | appC | cytochrome bd-II terminal oxidase subunit I |
| FTN_1621 | | FTT0089 | 28.98 | 1361.59 | 47 | FTN_1621 | predicted NAD/FAD-dependent oxidoreductase |
| FTN_1708 | | FTT1729 | 21.14 | 161.73 | 8 | FTN_1708 | ATP-binding cassette (ABC) superfamily protein |
| FTN_1718 | | FTT1739 | 144.83 | 1060.55 | 7 | kdpA | K(+)-ATPase uptake protein |
| FTN_1719 | | FTT1741 | 67.23 | 661.94 | 10 | FTN_1719 | D-isomer specific 2-hydroxyacid dehydrogenase |
| FTN_1733 | | FTT1779 | 31.17 | 430.02 | 14 | FTN_1733, | nicotinamide ribonucleoside (NR) uptake permease (PnuC) family protein |
| FTN_1738 | | FTT1774 | 38.16 | 493.67 | 13 | FTN_1738 | metallocarboxypeptidase |
| FTN_1755 | | FTT1757 | 63.02 | 377.85 | 6 | FTN_1755 | metabolite:H+ symporter (MHS) family protein |
|  | |  |  |  |  |  |  |
| **Gene is intact in U112 and its ortholog in Schu S4 is absent** | | | | | | | |
|  | | | | | | | |
| FTN_0004 | | None | 2.64 | 646.69 | 245 | FTN_0004 | aspartate/glutamate transporter |
| FTN_0005 | | None | 1.18 | 357.82 | 303 | corA | divalent inorganic cation transporter |
| FTN_0006 | | None | 2.07 | 385.09 | 186 | FTN_0006 | hypothetical protein |
| FTN_0007 | | None | 2.79 | 1859.31 | 667 | FTN_0007 | hypothetical protein |
| FTN_0008 | | None | 1.72 | 719.31 | 418 | FTN_0008 | 10 TMS drug/metabolite exporter protein |
| FTN_0017 | | None | 18.27 | 119.18 | 7 | FTN_0017 | phage integrase |
| FTN_0025 | | None | 1.03 | 303.86 | 296 | FTN_0025 | hypothetical protein |
| FTN_0038 | | None | 1.54 | 128.22 | 83 | FTN_0038 | hypothetical protein |
| FTN_0039 | | None | 6.94 | 435.64 | 63 | FTN_0039 | hypothetical protein |
| FTN_0040 | | None | 1.94 | 339.52 | 175 | FTN_0040 | hypothetical protein |
| FTN_0041 | | None | 1.78 | 440.74 | 248 | FTN_0041 | hypothetical protein |
| FTN_0042 | | None | 1 | 378.08 | 378 | FTN_0042 | hypothetical protein |
| FTN_0043 | | None | 37.83 | 604.07 | 16 | FTN_0043 | hypothetical protein |
| FTN_0044 | | None | 23.59 | 1140.76 | 48 | FTN_0044 | hypothetical protein |
| FTN_0045 | | None | 4.99 | 353.35 | 71 | FTN_0045 | hypothetical protein |
| FTN_0046 | | None | 16.52 | 407.95 | 25 | FTN_0046 | hypothetical protein |
| FTN_0047 | | None | 5.73 | 363.85 | 63 | FTN_0047 | hypothetical protein |
| FTN_0048 | | None | 1.2 | 408.31 | 341 | FTN_0048 | hypothetical protein |
| FTN_0049 | | None | 3.11 | 408.92 | 132 | FTN_0049 | hypothetical protein |
| FTN_0050 | | None | 10.35 | 441.99 | 43 | FTN_0050 | hypothetical protein |
|  | |  |  |  |  |  |  |

(Continued)

**S2 Table (continued).**

|  |  | | |  |  |  |  | |  |
| --- | --- | --- | --- | --- | --- | --- | --- | --- | --- |
| **Locus in U112** | **Locus in**  **Schu S4** | | | **Intensity in Schu S4** | **Intensity in U112** | **Fold Difference** | **Gene in U112** | | **Product** |
|  |  | | |  |  |  |  | |  |
|  |  | | |  |  |  |  | |  |
| FTN_0051 | | | None | 14.87 | 1059.99 | 71 | | FTN_0051 | hypothetical protein |
| FTN_0052 | | | None | 1.98 | 451.66 | 228 | | FTN_0052 | hypothetical protein |
| FTN_0053 | | | None | 3.74 | 503.88 | 135 | | FTN_0053 | hypothetical protein |
| FTN_0054 | | | None | 1.1 | 530.19 | 484 | | FTN_0054 | hypothetical protein |
| FTN_0055 | | | None | 2.47 | 329.85 | 134 | | tyrA | prephenate dehydrogenase |
| FTN_0059 | | | None | 1 | 433.65 | 434 | | leuB | 3-isopropylmalate dehydrogenase |
| FTN_0060 | | | None | 1.51 | 659.2 | 438 | | leuD | isopropylmalate isomerase small subunit |
| FTN_0061 | | | None | 20.38 | 487.47 | 24 | | leuC | isopropylmalate isomerase |
| FTN_0115 | | | None | 16.5 | 121.96 | 7 | | FTN_0115 | Na+/H+ antiporter |
| FTN_0130 | | | None | 8.99 | 309.59 | 34 | | FTN_0130 | glycosyl transferase |
| FTN_0215 | | | None | 1.89 | 149.15 | 79 | | FTN_0215 | hypothetical protein |
| FTN_0267 | | | None | 1.63 | 670.02 | 411 | | FTN_0267 | hypothetical protein |
| FTN_0282 | | | None | 2.21 | 647.13 | 292 | | FTN_0282 | hypothetical protein |
| FTN_0309 | | | None | 5.2 | 323.76 | 62 | | FTN_0309 | hypothetical membrane protein |
| FTN_0367 | | | None | 5.24 | 96.74 | 18 | | FTN_0367 | phage integrase |
| FTN_0369 | None | | | 4.96 | 311.36 | 63 | FTN_0369 | | hypothetical protein |
| FTN_0370 | None | | | 3.57 | 479.7 | 134 | FTN_0370 | | hypothetical protein |
| FTN_0371 | None | | | 21.76 | 179.27 | 8 | FTN_0371 | | hypothetical protein |
| FTN_0415 | None | | | 2.07 | 3254.27 | 1569 | pilA | | Type IV pili, pilus assembly protein |
| FTN_0418 | None | | | 1.63 | 809.72 | 497 | FTN_0418 | | endonuclease |
| FTN_0451 | None | | | 3.54 | 847.72 | 239 | FTN_0451 | | signal transduction protein with a PAS, a PAC, an EAL and a GGDEF domain |
| FTN_0452 | None | | | 24.08 | 284.75 | 12 | FTN_0452 | | hypothetical protein |
| FTN_0453 | None | | | 5.56 | 138.01 | 25 | FTN_0453 | | glycosyl transferase |
| FTN_0454 | None | | | 3.64 | 135.46 | 37 | FTN_0454 | | hypothetical protein |
| FTN_0455 | None | | | 2.7 | 583.03 | 216 | FTN_0455 | | CheB methylesterase/CheR methyltransferase |
| FTN_0456 | None | | | 7.18 | 339.3 | 47 | FTN_0456 | | signal transduction protein with a PAS, a PAC, an EAL and a GGDEF domain |
| FTN_0509 | None | | | 17.62 | 308.72 | 18 | FTN_0509 | | hypothetical protein |
| FTN_0510 | None | | | 4.76 | 192.69 | 40 | FTN_0510 | | hypothetical protein |
| FTN_0587 | None | | | 2.63 | 424.34 | 161 | FTN_0587 | | deoxyguanosinetriphosphate triphosphohydrolase |
| FTN_0617 | None | | | 8.45 | 915.86 | 108 | FTN_0617 | | ROK family protein |
| FTN_0618 | None | | | 12.46 | 826.51 | 66 | FTN_0618 | | ROK family protein |
| FTN_0703 | None | | | 8.51 | 173.53 | 20 | FTN_0703 | | type I restriction-modification system, subunit S |
|  | |  | | | | | | | |

(Continued)

**S2 Table (continued).**

|  |  |  |  |  |  |  | |
| --- | --- | --- | --- | --- | --- | --- | --- |
| **Locus in U112** | **Locus in**  **Schu S4** | **Intensity in Schu S4** | **Intensity in U112** | **Fold Difference** | **Gene in U112** | **Product** | |
|  |  |  |  |  |  |  | |
|  |  |  |  |  |  |  | |
| FTN_0704 | None | 1.35 | 277.72 | 206 | FTN_0704 | type I restriction-modification system, subunit M (methyltransferase) | |
| FTN_0705 | None | 3.82 | 262.39 | 69 | FTN_0705 | abortive infection bacteriophage resistance protein | |
| FTN_0706 | None | 7.58 | 261.24 | 34 | FTN_0706 | hypothetical membrane protein | |
| FTN_0707 | None | 3.66 | 369.69 | 101 | FTN_0707 | type I restriction-modification system, subunit S | |
| FTN_0708 | None | 7.73 | 245.52 | 32 | FTN_0708 | hypothetical protein | |
| FTN_0709 | None | 1.92 | 146.88 | 77 | FTN_0709 | hypothetical protein | |
| FTN_0710 | None | 4.35 | 302.03 | 69 | FTN_0710 | type I restriction-modification system, subunit R (restriction) | |
| FTN_0711 | None | 6.37 | 270.9 | 43 | FTN_0711 | metal-dependent hydrolase | |
| FTN_0730 | None | 1.94 | 631.99 | 326 | acs | acyl-coenzyme A synthetase/AMP-(fatty) acid ligases | |
| FTN_0761 | None | 10.96 | 999.64 | 91 | FTN_0761 | radical SAM family protein | |
| FTN_0762 | None | 1.14 | 899.46 | 786 | grxC | glutaredoxin like protein | |
| FTN_0763 | None | 2.68 | 680.05 | 254 | FTN_0763 | thioesterase superfamily protein | |
| FTN_0808 | None | 33.18 | 319.7 | 10 | FTN_0808 | acetoacetate decarboxylase | |
| FTN_0825 | None | 12.28 | 852.11 | 69 | FTN_0825 | aldo/keto reductase family protein | |
| FTN_0837 | None | 7.64 | 353.36 | 46 | FTN_0837 | hypothetical protein | |
| FTN_0864 | None | 3.83 | 2558.15 | 668 | FTN_0864 | hypothetical protein | |
| FTN_0928 | None | 4.87 | 239.18 | 49 | cysD | sulfate adenylyltransferase subunit 2 | |
| FTN_0929 | None | 2.37 | 257.74 | 109 | FTN_0929 | hypothetical protein | |
| FTN_0930 | None | 39.68 | 559.64 | 14 | FTN_0930 | hypothetical protein | |
| FTN_0931 | None | 4.5 | 428.27 | 95 | FTN_0931 | hypothetical protein | |
| FTN_0932 | None | 1 | 331.23 | 331 | FTN_0932 | ABC transporter,  ATP-binding protein | |
| FTN_0933 | None | 2.62 | 365.07 | 140 | FTN_0933 | hypothetical protein | |
| FTN_0934 | None | 3.49 | 386.06 | 111 | FTN_0934 | hypothetical protein | |
| FTN_0935 | None | 6.25 | 260.5 | 42 | asnB | asparagine synthase | |
| FTN_0936 | None | 1.26 | 182.44 | 145 | FTN_0936 | hypothetical protein | |
| FTN_0937 | None | 2.93 | 206.31 | 70 | FTN_0937 | hypothetical protein | |
| FTN_0938 | None | 1 | 914.79 | 915 | FTN_0938 | hypothetical protein | |
| FTN_0939 | None | 3.95 | 1760.31 | 445 | FTN_0939 | hypothetical protein | |
| FTN_0965 | None | 1.53 | 238.99 | 156 | FTN_0965 | exopeptidase |  |
| FTN_0969 | None | 29.25 | 224.31 | 8 | FTN_0969 | hypothetical protein | |
| FTN_1015 | None | 1.22 | 612.66 | 502 | FTN_1015 | isochorismatase family protein | |
| FTN_1079 | None | 33.63 | 798.5 | 24 | FTN_1079 | sugar porter (SP) family | |
| FTN_1101 | None | 24.57 | 290.04 | 12 | FTN_1101 | hypothetical protein | |
|  |  |  |  |  |  |  | |

(Continued)

**S2 Table (continued).**

|  |  |  |  |  |  |  | |
| --- | --- | --- | --- | --- | --- | --- | --- |
| **Locus in U112** | **Locus in**  **Schu S4** | **Intensity in Schu S4** | **Intensity in U112** | **Fold Difference** | **Gene in U112** | **Product** | |
|  |  |  |  |  |  |  | |
|  |  |  |  |  |  |  | |
| FTN_1102 | None | 2.04 | 336.49 | 165 | FTN_1102 | hypothetical protein | |
| FTN_1103 | None | 3.13 | 523.53 | 167 | FTN_1103 | hypothetical protein | |
| FTN_1104 | None | 23.16 | 660.25 | 29 | FTN_1104 | hypothetical protein | |
| FTN_1153 | None | 1.83 | 155.99 | 85 | FTN_1153 | hypothetical protein | |
| FTN_1154 | None | 2.69 | 285.76 | 106 | FTN_1154 | restriction-modification system, subunit S | |
| FTN_1215 | None | 1.15 | 696.92 | 605 | kpsC | capsule polysaccharide export protein KpsC | |
| FTN_1216 | None | 4.92 | 563.62 | 115 | FTN_1216 | hypothetical protein | |
| FTN_1216 | None | 60.56 | 647.7 | 11 | FTN_1216 | hypothetical protein | |
| FTN_1230 | None | 2.49 | 2648.04 | 1063 | FTN_1230 | hypothetical protein | |
| FTN_1261 | None | 1.09 | 401.29 | 368 | FTN_1261 | hypothetical protein | |
| FTN_1378 | None | 35.65 | 426.06 | 12 | FTN_1378 | hypothetical protein | |
| FTN_1397 | None | 13.72 | 813.2 | 59 | FTN_1397 | hypothetical protein | |
| FTN_1420 | None | 1 | 1352.75 | 1353 | wzx | O antigen flippase | |
| FTN_1422 | None | 1.12 | 621.34 | 557 | wbtN | glycosyl transferase | |
| FTN_1424 | None | 3.37 | 2229.52 | 662 | FTN_1424 | hypothetical membrane protein | |
| FTN_1428 | None | 8.37 | 1841.02 | 220 | wbtO | transferase | |
| FTN_1429 | None | 34.87 | 2067.64 | 59 | wbtP | galactosyl transferase | |
| FTN_1430 | None | 9.17 | 465.08 | 51 | wbtQ | aminotransferase | |
| FTN_1488 | None | 1.6 | 534.72 | 334 | FTN_1488 | prophage maintenance system killer protein (DOC) | |
| FTN_1489 | None | 2.52 | 337.84 | 134 | FTN_1489 | hypothetical protein | |
| FTN_1490 | None | 6.37 | 1147.9 | 180 | FTN_1490 | hypothetical protein | |
| FTN_1698 | None | 9.09 | 155.97 | 17 | FTN_1698 | Dam-replacing family protein | |
| FTN_1727 | None | 15.37 | 103.63 | 7 | dapD | tetrahydrodipicolinate succinylase subunit | |
| FTN_1728 | None | 5.93 | 136.88 | 23 | dapA | dihydrodipicolinate synthase | |
| FTN_1729 | None | 32.28 | 219.04 | 7 | dapB | dihydrodipicolinate reductase | |
| FTN_1730 | None | 11.51 | 1220.67 | 106 | lysC | aspartate kinase III | |
| FTN_1731 | None | 7.3 | 374.33 | 51 | pip | proline iminopeptidase | |
| FTN_1732 | None | 1.29 | 578.58 | 450 | FTN_1732 | Mg-dependent DNase | |
| FTN_1756 | None | 9.6 | 540.56 | 56 | bcp | bacterioferritin comigratory protein | |
| FTN_1757 | None | 11.37 | 409.83 | 36 | FTN_1757 | D-isomer specific 2-hydroxyacid dehydrogenase | |
| FTN_1759 | None | 1.28 | 583.36 | 457 | FTN_1759 | hypothetical protein | |
| FTN_1766 | None | 13.96 | 582.51 | 42 | FTN_1766 | drug/metabolite transporter (DMT) superfamily protein | |
| FTN_1767 | None | 2.04 | 336.49 | 165 | rbsK | ribokinase, pfkB family | |
|  |  |  |  |  |  |  | |
| **Gene is pseudogene in U112 and its ortholog in Schu S4 is absent** | | | | | | |  |
|  |  |  |  |  |  |  | |
| FTN_0283 | None | 4.42 | 169.11 | 38 | FTN_0283 | None | |
| FTN_1379 | None | 3.82 | 197.02 | 52 | FTN_1379 | None | |
|  |  |  |  |  |  |  | |
