# Supplementary material for: Comparative Transcriptional Analyses of Francisella tularensis and Francisella novicida
Source: PLoS One. 2016 Aug 18;11(8):e0158631. doi: 10.1371/journal.pone.0158631 (PMC4990168; doi:10.1371/journal.pone.0158631)
Supplement: S3 Table — (DOCX) [file pone.0158631.s003.docx]

**S3 Table:** **ORFs with high expression (≥ 5 fold and p≤ 0.05) in Ftt compared to Fth.**

|  |  |  |  |  |  |  |
| --- | --- | --- | --- | --- | --- | --- |
| **Locus in Schu S4** | **Locus in OR960246*** | **Intensity in Schu S4** | **Intensity in OR960246** | **Fold Difference** | **Gene in Schu S4** | **Product** |
|  |  |  |  |  |  |  |
|  |  |  |  |  |  |  |
| **Gene is intact in Schu S4 and its ortholog in OR960246 is also intact** | | | | | | |
|  |  |  |  |  |  |  |
| FTT0127 | FTH_1591 | 222.84 | 41.31 | 5 | FTT0127 | major facilitator superfamily (MFS) transport protein |
| FTT0442 | FTH_1570 | 501.84 | 65.04 | 8 | FTT0442 | major facilitator superfamily (MFS) transport protein |
| FTT0707 | FTH_1479 | 708.49 | 121.12 | 6 | FTT0707 | nicotinamide mononucleotide transport (NMT) family protein |
| FTT0784 | FTH_1400 | 194.22 | 34.8 | 6 | FTT0784 | hypothetical protein |
| FTT0815 | FTH_1370 | 150.95 | 27.86 | 5 | FTT0815 | chitin binding protein |
| FTT1004 | FTH_1172 | 278.56 | 49.54 | 6 | FTT1004 | DMT superfamily drug/metabolite transporter |
| FTT1089 | FTH_1087 | 1614.92 | 312.76 | 5 | FTT1089 | isochorismatase hydrolase family protein |
| FTT1090 | FTH_1086 | 1965.15 | 250.54 | 8 | FTT1090 | PnuC |
| FTT1175 | FTH_0772 | 142.58 | 25.97 | 5 | FTT1175 | hypothetical protein |
| FTT1234 | FTH_0713 | 269.99 | 15.05 | 18 | FTT1234 | choloylglycine hydrolase family protein |
| FTT1414 | FTH_0648 | 456.75 | 63.87 | 7 | FTT1414 | hypothetical protein |
| FTT1784 | FTH_1866 | 364.83 | 34.65 | 11 | FTT1784 | hypothetical protein |
|  |  |  |  |  |  | |
| **Gene is intact in Schu S4 and its ortholog in OR960246 is a pseudogene** | | | | | | |
|  |  |  |  |  |  |  |
| FTT0017 | FTH_1780 | 640.79 | 95.05 | 7 | FTT0017 | ATP-binding cassette (ABC) superfamily protein |
| FTT0018 | FTH_1779 | 563.8 | 63.61 | 9 | FTT0018 | RND efflux membrane fusion protein |
| FTT0088 | FTH_1707 | 1215.75 | 233.52 | 5 | pilT | Type IV pili nucleotide-binding protein |
| FTT0095 | FTH_1700 | 984.93 | 27.48 | 36 | FTT0095 | hypothetical protein |
| FTT0308 | FTH_0215 | 160.05 | 18.5 | 9 | FTT0308 | hypothetical protein |
| FTT0425 | FTH_0492 | 341.85 | 20.04 | 17 | asd | aspartate semialdehyde dehydrogenase |
| FTT0432 | FTH_0499 | 316.49 | 44.3 | 7 | speA | arginine decarboxylase |
| FTT0433 | FTH_0499 | 557.88 | 48.6 | 11 | FTT0433 | hypothetical protein |
| FTT0435 | FTH_0502 | 468.94 | 40.55 | 12 | FTT0435 | Carbon-nitrogen hydrolase family protein |
| FTT0447 | FTH_1565 | 337.77 | 47.28 | 7 | FTT0447c | hypothetical protein |
| FTT0475 | FTH_1534 | 183.95 | 23.51 | 8 | msc | mechanosensitive ion channel protein |
|  | | | | | | |

(Continued)

**S3 Table (continued).**

|  |  |  |  |  |  |  |
| --- | --- | --- | --- | --- | --- | --- |
| **Locus in Schu S4** | **Locus in OR960246*** | **Intensity in Schu S4** | **Intensity in OR960246** | **Fold Difference** | **Gene in Schu S4** | **Product** |
|  |  |  |  |  |  |  |
|  |  |  |  |  |  |  |
| FTT0525 | FTH_0957 | 174.64 | 27.76 | 6 | FTT0525 | hypothetical protein |
| FTT0783 | FTH_1401 | 362.34 | 36.61 | 10 | ars | Arylsulphatase |
| FTT0785 | FTH_1399 | 110.04 | 14.87 | 7 | FTT0785 | hypothetical protein |
| FTT0829 | FTH_0321 | 321.3 | 63.82 | 5 | FTT0829 | aspartate:alanine exchanger (AAE) family protein |
| FTT0889 | FTH_0383 | 995.16 | 131.26 | 8 | FTT0889 | pilus assembly protein (pilA) |
| FTT0992 | FTH_1184 | 584.5 | 89.65 | 7 | FTT0992 | small conductance mechanosensitive ion channel (MscS) family protein |
| FTT0995 | FTH_1181 | 178.31 | 22.6 | 8 | FTT0995 | major facilitator superfamily (MFS) transport protein |
| FTT1020 | FTH_1045 | 106.67 | 12.46 | 9 | FTT1020 | amino acid permease |
| FTT1091 | FTH_1084 | 663.11 | 56.54 | 12 | FTT1091 | isochorismatase hydrolase family protein |
| FTT1126 | FTH_0825 | 779.25 | 136.57 | 6 | FTT1126 | aromatic amino acid transporter of the HAAAP family |
| FTT1233 | FTH_0714 | 223.85 | 37.48 | 6 | yjdL | proton-dependent oligopeptide transporter (POT) family protein, di- or tripeptide:H+ symporter |
| FTT1383 | FTH_1106 | 951.14 | 85.09 | 11 | sun | tRNA and rRNA cytosine-C5-methylases, sun protein |
| FTT1419 | FTH_0643 | 172.1 | 31.25 | 6 | FTT1419 | hypothetical protein |
| FTT1536 | FTH_0577 | 1012.48 | 59.96 | 17 | FTT1536 | dimethylarginine dimethylaminohydrolase |
| FTT1628 | FTH_1635 | 235.47 | 36.63 | 6 | FTT1628 (TlyC) | transporter-associated protein, HlyC/CorC family |
| FTT1651 | FTH_0041 | 138.35 | 20.66 | 7 | FTT1651 | hypothetical protein |
| FTT1715 | FTH_1128 | 1524.35 | 128.82 | 12 | pdpD2 | hypothetical protein |
| FTT1744 | FTH_0006 | 322.95 | 54.8 | 6 | ipdC | indolepyruvate decarboxylase |
| FTT1789 | FTH_1871 | 322.52 | 35.82 | 9 | FTT1789 | hypothetical protein |
|  | | | | | | |
| **Gene is intact in Schu S4 and its ortholog in OR960246 is absent** | | | | | | |
|  |  |  |  |  |  |  |
| FTT0126 | None | 19.3 | 3.4 | 6 | oppF | oligopeptide transporter, subunit F |
| FTT0382 | None | 478.53 | 24.29 | 20 | FTT0382 | hypothetical protein |
| FTT0446 | None | 772.23 | 8.17 | 95 | FTT0446 | POT family protein |
| FTT0553 | None | 281.13 | 10.87 | 26 | FTT0553 | hypothetical protein |
| FTT0960 | None | 1326.58 | 24.55 | 54 | FTT0960 | hypothetical protein |
| FTT0961 | None | 1075.23 | 55.44 | 19 | mdaB | modulator of drug activity B |
|  |  |  |  |  |  |  |

(Continued)

**S3 Table (continued).**

|  |  |  |  |  |  |  | |
| --- | --- | --- | --- | --- | --- | --- | --- |
| **Locus in Schu S4** | **Locus in OR960246*** | **Intensity**  **in Schu S4** | **Intensity in OR960246** | **Fold Difference** | **Gene in Schu S4** | **Product** | |
|  |  |  |  |  |  |  | |
|  |  |  |  |  |  |  | |
| FTT0962 | None | 650.45 | 7.68 | 85 | FTT0962 | ThiJ/PfpI family protein | |
| FTT1005 | None | 514.35 | 7.79 | 66 | yhiP | Proton-dependent oligopeptide transporter (POT) family protein | |
| FTT1068 | None | 263.32 | 6.22 | 42 | FTT1068 | hypothetical protein | |
| FTT1069 | None | 183.92 | 25.78 | 7 | FTT1069 | hypothetical protein | |
| FTT1072 | None | 334.14 | 10.22 | 33 | FTT1072 | hypothetical protein | |
| FTT1242 | None | 3253.89 | 9.43 | 345 | FTT1242 | hypothetical protein | |
| FTT1266 | None | 130.67 | 3.67 | 36 | yhhW | pirin family protein | |
| FTT1308 | None | 344.75 | 15.65 | 22 | FTT1308 | hypothetical protein | |
| FTT1580 | None | 710.46 | 7.54 | 94 | FTT1580 | hypothetical protein | |
| FTT1791 | None | 2883.33 | 21.85 | 132 | FTT1791 | hypothetical protein | |
|  |  |  |  |  |  |  | |
| **Gene is a pseudogene in Schu S4 and its ortholog in OR960246 is absent** | | | | | | |  |
|  |  |  |  |  |  |  | |
| FTT0441 | FTH_1571 | 243.34 | 41.04 | 6 | FTT0441 | None | |
| FTT0672 | FTH_0925 | 1466.81 | 165.68 | 9 | FTT0672 | None | |
| FTT0828 | FTH_0320 | 222.7 | 30.75 | 7 | FTT0828 | None | |
| FTT1501 | FTH_0292 | 954.76 | 155.98 | 6 | FTT1501 | None | |
|  |  |  |  |  |  |  | |
| **Gene is pseudogene in Schu S4 and its ortholog in OR960246 is also pseudogene** | | | | | | |  |
|  |  |  |  |  |  |  | |
| FTT0426 | FTH_0493 | 382.77 | 39.23 | 10 | thrA | None | |
| FTT0427 | FTH_0494 | 128.29 | 11.25 | 11 | thrB | None | |
| FTT0498 | FTH_1510 | 483.13 | 40.76 | 12 | FTT0498 | None | |
| FTT0539 | FTH_0970 | 388.9 | 9.27 | 42 | FTT0539 | None | |
| FTT0672 | FTH_0925 | 239.5 | 38.43 | 6 | FTT0672 | None | |
| FTT0830 | FTH_0322 | 842.65 | 117.26 | 7 | FTT0830 | None | |
| FTT0949 | FTH_1233 | 1561.13 | 286.41 | 5 | FTT0949 | None | |
| FTT1139 | FTH_0811 | 86.23 | 14.82 | 6 | FTT1139 | None | |
| FTT1482 | FTH_0313 | 305.93 | 47.91 | 6 | FTT1482 | None | |
| FTT1544 | FTH_0567 | 518.61 | 60.25 | 9 | FTT1544 | None | |
|  |  |  |  |  |  |  | |
| **Gene is a pseudogene in Schu S4 and its ortholog in OR960246 is absent** | | | | | | |  |
|  |  |  |  |  |  |  | |
| FTT0706 | None | 1143.26 | 56.98 | 20 | FTT0706 | None | |
| FTT0921 | None | 119.79 | 9.7 | 12 | FTT0921 | None | |
| FTT1070 | None | 269.07 | 8.96 | 30 | FTT1070 | None | |
| FTT1582 | None | 522.77 | 11.07 | 47 | FTT1582 | None | |
| FTT1583 | None | 190.74 | 15.84 | 12 | FTT1583 | None | |
| FTT1670 | None | 131.7 | 3.15 | 42 | FTT1670 | None | |
| FTT1716 | None | 743.67 | 33.35 | 22 | FTT1716 | None | |
| FTT1757 | None | 63.02 | 5.89 | 11 | FTT1757 | None | |
| FTT1790 | None | 118.99 | 12.63 | 9 | FTT1790 | None | |
|  |  |  |  |  |  |  | |
