# Supplementary material for: Comparative Transcriptional Analyses of Francisella tularensis and Francisella novicida
Source: PLoS One. 2016 Aug 18;11(8):e0158631. doi: 10.1371/journal.pone.0158631 (PMC4990168; doi:10.1371/journal.pone.0158631)
Supplement: S4 Table — (DOCX) [file pone.0158631.s004.docx]

S4 Table: ORFs with high expression (≥ 5 fold and p≤ 0.05) in Fth compared to Ftt.

|  |  |  |  |  |  |  | |
| --- | --- | --- | --- | --- | --- | --- | --- |
| **Locus in OR960246** | **Locus in**  **Schu S4*** | **Intensity**  **in Schu S4** | **Intensity in OR960246** | **Fold Difference** | **Gene in OR960246** | **Product** | |
|  |  |  |  |  |  |  | |
|  |  |  |  |  |  |  | |
| **Gene is intact in OR960246 and its ortholog is also intact in Schu S4** | | | | | | |  |
|  |  |  |  |  |  |  | |
| FTH_0338 | FTT0845 | 6.94 | 61.07 | 9 | FTH_0338 | hypothetical protein | |
| FTH_0806 | FTT1143 | 33.77 | 486.05 | 14 | FTH_0806 | hypothetical protein | |
| FTH_0895 | FTT0642 | 192.4 | 1123.47 | 6 | ilvH | acetolactate synthase small subunit | |
| FTH_1669 | FTT0158 | 13.64 | 117.3 | 9 | FTH_1669 | hypothetical membrane protein | |
| **Gene is a pseudogene in OR960246 and its ortholog in Schu S4 is an intact gene** | | | | | | |  |
|  |  |  |  |  |  |  | |
| FTH_0335 | FTT0844 | 26.05 | 291.93 | 11 | FTH_0335 | short-chain alcohol dehydrogenase-like dehydrogenase | |
| FTH_0374 | FTT0880 | 84.96 | 447.56 | 5 | FTH_0374 | sterol desaturase | |
| FTH_0896 | FTT0643 | 50.52 | 702.25 | 14 | ilvC | ketol-acid reductoisomerase | |
| FTH_1180 | FTT0996 | 13.48 | 71.81 | 5 | FTH_1180 | cardiolipin synthase | |
| FTH_1515 | FTT0493 | 12.28 | 119.46 | 10 | araJ | major facilitator superfamily (MFS) transport protein | |
| FTH_1625 | FTT1619 | 31.73 | 203.04 | 6 | FTH_1625 | acetyltransferase | |
|  |  |  |  |  |  |  | |
| **Gene is intact in OR960246 and its ortholog in Schu S4 is absent** | | | | | | |  |
|  |  |  |  |  |  |  | |
| FTH_0048 | None | 2.47 | 134.96 | 55 | tyrA | prephenate dehydrogenase | |
| FTH_0467 | None | 1 | 452.29 | 452 | FTH_0467 | hypothetical protein | |
| FTH_0807 | None | 7.77 | 367.84 | 47 | FTH_0807 | hypothetical protein | |
| FTH_1195 | None | 3.83 | 796.1 | 208 | FTH_1195 | hypothetical protein | |
| FTH_1813 | None | 1.51 | 13.86 | 9 | leuD | isopropylamate dehydratase | |
|  |  |  |  |  |  |  | |
| **Gene is a pseudogene in OR960246 and its ortholog in Schu S4 is absent** | | | | | | |  |
|  |  |  |  |  |  |  | |
| FTH_0704 | None | 8.08 | 51.21 | 6 | FTH_0704 | None | |
| FTH_0915 | None | 16.08 | 198.26 | 12 | FTH_0915 | None | |
|  |  |  |  |  |  |  | |
| **Gene is a Pseudogene in both the Strains** | | | | | | |  |
|  |  |  |  |  |  |  | |
| FTH_0752 | FTT1195c | 116.15 | 936.81 | 8 | FTH_0752 | None | |
| FTH_0805 | FTT1144 | 32.05 | 211.74 | 7 | FTH_0805 | None | |
| FTH_0816 | FTT1135c | 56.9 | 343.72 | 6 | FTH_0816 | None | |
| FTH_0894 | FTT0641 | 115.81 | 744.44 | 6 | FTH_0894 | None | |
|  |  |  |  |  |  |  | |
