# Supplementary material for: Comparative Transcriptional Analyses of Francisella tularensis and Francisella novicida
Source: PLoS One. 2016 Aug 18;11(8):e0158631. doi: 10.1371/journal.pone.0158631 (PMC4990168; doi:10.1371/journal.pone.0158631)
Supplement: S5 Table — (DOCX) [file pone.0158631.s005.docx]

S5 Table: Genes with High Expression (≥ 5 fold and p≤ 0.05) in *Fth* Compared to *Fn.*

|  |  |  |  |  |  |  | |
| --- | --- | --- | --- | --- | --- | --- | --- |
| **Locus in OR960246** | **Locus in U112** | **Intensity in OR960246** | **Intensity in U112** | **Fold Difference** | **Gene** | **Product** | |
|  |  |  |  |  |  |  | |
|  |  |  |  |  |  |  | |
| **Gene is intact in OR960246 and its ortholog in U112 is also intact** | | | | | | | |
|  | | | | | | | |
| FTH_0037 | FTN_0028 | 1235.54 | 13.92 | 89 | FTH_0037 | hypothetical protein | |
| FTH_0038 | FTN_0029 | 1237.8 | 7.85 | 158 | FTH_0038 | probable multidrug efflux pump | |
| FTH_0039 | FTN_0030 | 3363.77 | 23.25 | 145 | FTH_0039 | hypothetical protein | |
| FTH_0209 | FTN_0217 | 282.54 | 38.78 | 7 | lldD | L-lactate dehydrogenase | |
| FTH_0212 | FTN_0220 | 599.6 | 134.73 | 4 | fumC | fumarate hydratase | |
| FTH_0676 | FTN_1353 | 1348.5 | 229.42 | 6 | panC | pantoate--beta-alanine ligase | |
| FTH_0758 | FTN_1167 | 2444.87 | 381.67 | 6 | FTH_0758 | hypothetical protein | |
| FTH_1768 | FTN_1682 | 275.91 | 10.37 | 27 | frgA | possible siderophore biosynthesis IucA / IucC family protein | |
| FTH_1769 | FTN_1683 | 100.66 | 16.25 | 6 | FTH_1769 | possible MFS family major facilitator transporter, multidrug resistance protein | |
| FTH_1770 | FTN_1684 | 117.87 | 6.76 | 17 | lysA2 | diaminopimelate decarboxylase | |
| FTH_1883 | FTN_1778 | 2120.01 | 476.04 | 4 | trpE2 | anthranilate synthase component I | |
|  |  |  |  |  |  |  | |
| **Gene is intact in OR960246 and its ortholog in U112 is absent** | | | | | | | |
|  | | | | | | | |
| FTH_0033 | None | 2257.58 | 8.96 | 252 | FTH_0033 | | hypothetical protein |
| FTH_0206 | None | 690.86 | 2.25 | 307 | FTH_0206 | | hypothetical protein |
| FTH_0207 | None | 289.23 | 1 | 289 | FTH_0207 | | hypothetical protein |
| FTH_0384 | None | 2307.36 | 32.36 | 71 | FTH_0384 | | type IV pili fiber protein |
| FTH_0458 | None | 709.76 | 11.54 | 61 | FTH_0458 | | hypothetical protein |
| FTH_0497 | None | 299.91 | 22.57 | 13 | speH | | adenosylmethionine decarboxylase |
| FTH_0498 | None | 151.85 | 7.45 | 20 | speE | | spermidine synthase |
| FTH_0508 | None | 268.27 | 7.79 | 34 | FTH_0508 | | hypothetical protein |
| FTH_0529 | None | 236.39 | 2.79 | 85 | FTH_05299 | | possible exci/endonuclease |
| FTH_0531 | None | 332.66 | 16.51 | 20 | res | | deoxyribonuclease |
| FTH_0590 | None | 230.08 | 3.04 | 76 | hrpA | | ATP-dependent helicase HrpA |
| FTH_0593 | None | 2328.56 | 2.42 | 961 | FTH_0593 | | galactosyltransferase |
| FTH_0594 | None | 1862.09 | 27.57 | 68 | FTH_0594 | | UDP-glucose 4-epimerase |
| FTH_0595 | None | 1725.82 | 2.53 | 682 | FTH_0595 | | galacturonosyltransferase |
| FTH_0598 | None | 1383.79 | 1 | 1384 | wzy | | O-antigen polymerase |
| FTH_0599 | None | 1740.67 | 1.13 | 1537 | FTH_0599 | | glycosyltransferase |
| FTH_0602 | None | 1752.56 | 1.37 | 1276 | FTH_0602 | | probable formyltransferase |
| FTH_0603 | None | 550.2 | 1.36 | 404 | FTH_0603 | | PST family polysaccharide transporter |
| FTH_0604 | None | 1691.76 | 2.78 | 610 | FTH_0604 | | glycosyltransferase |
| FTH_0605 | None | 1295.3 | 10.05 | 129 | FTH_0605 | | glucose-1-phosphate thymidylyltransferase |
| FTH_0671 | None | 450.64 | 2.42 | 186 | FTH_0671 | | ATP-dependent DNA helicase |
|  |  |  |  |  |  | |  |

(Continued)

**S5 Table (continued).**

|  |  |  |  |  |  |  | |
| --- | --- | --- | --- | --- | --- | --- | --- |
| **Locus in OR960246** | **Locus in U112** | **Intensity in OR960246** | **Intensity in U112** | **Fold Difference** | **Gene** | **Product** | |
|  |  |  |  |  |  |  | |
|  |  |  |  |  |  |  | |
| FTH_0760 | None | 99.8 | 1.42 | 70 | FTH_0760 | hypothetical protein | |
| FTH_0807 | None | 367.84 | 6.72 | 55 | FTH_0807 | hypothetical protein | |
| FTH_0853 | None | 1513.67 | 14.89 | 102 | FTH_0853 | MFS family major facilitator transporter | |
| FTH_0856 | None | 226.87 | 4.01 | 57 | FTH_0856 | hypothetical protein | |
| FTH_0955 | None | 262.7 | 9.91 | 26 | FTH_0955 | type I site-specific deoxyribonuclease | |
| FTH_1095 | None | 196.26 | 1.37 | 144 | FTH_1095 | hypothetical protein | |
| FTH_1097 | None | 187.72 | 5.76 | 33 | FTH_1097 | hypothetical protein | |
| FTH_1222 | None | 397.23 | 2.73 | 145 | FTH_1222 | dehdyrogenase | |
| FTH_1303 | None | 134.03 | 1.45 | 92 | alr | alanine racemase | |
| FTH_1329 | None | 1395.53 | 5.3 | 263 | FTH_1329 | probable transcriptional regulator | |
| FTH_1388 | None | 852.57 | 1.18 | 722 | FTH_1388 | hypothetical protein | |
| FTH_1389 | None | 1081.95 | 2.68 | 404 | FTH_1389 | hypothetical protein | |
| FTH_1390 | None | 923.7 | 3.38 | 273 | FTH_1390 | | nucleotidyltransferase |
| FTH_1574 | None | 132.36 | 21.93 | 6 | FTH_1574 | | hypothetical protein |
| FTH_1577 | None | 115.78 | 2.68 | 43 | FTH_1577 | | hypothetical protein |
| FTH_1578 | None | 466.84 | 16.34 | 29 | FTH_1578 | | LysR family transcriptional regulator |
| FTH_1781 | None | 2253.31 | 1 | 2253 | FTH_1781 | | hypothetical protein |
| FTH_1816 | None | 3652.46 | 3.25 | 1124 | FTH_1816 | | hypothetical protein |
| FTH_1843 | None | 234.49 | 1 | 234 | FTH_1843 | | YGGT family membrane protein |
|  |  |  |  |  |  | |  |
| **Gene is pseudogene in OR960246 and its ortholog in U112 is intact** | | | | | | | |
|  |  |  |  |  |  | |  |
| FTH_0829 | FTN_1103 | 383.98 | 16.08 | 24 | FTH_0829 | | None |
| FTH_1337 | FTN_0715 | 747.92 | 51.28 | 15 | FTH_1337 | | None |
|  |  |  |  |  |  | |  |
| **Gene is pseudogene in OR960246 and its ortholog in U112 is also a pseudogene** | | | | | | | |
|  |  |  |  |  |  | |  |
| FTH_0607 | FTN_1419 | 1456.09 | 9.92 | 147 | FTH_0607 | | None |
|  |  |  |  |  |  | |  |
| **Gene is pseudogene in OR960246 and its ortholog in U112 is absent** | | | | | | | |
|  |  |  |  |  |  | |  |
| FTH_0026 | None | 2073.93 | 1.12 | 1855 | FTH_0026 | | None |
| FTH_0273 | None | 942.66 | 1.25 | 753 | FTH_0273 | | None |
| FTH_0607 | None | 1439.21 | 16.27 | 88 | FTH_0607 | | None |
| FTH_0829 | None | 419.2 | 3.53 | 119 | FTH_0829 | | None |
| FTH_0855 | None | 334.09 | 3.51 | 95 | FTH_0855 | | None |
| FTH_0930 | None | 756.06 | 1 | 756 | FTH_0930 | | None |

(Continued)

**S5 Table (continued).**

|  |  | |  | |  | |  | |  |  | |
| --- | --- | --- | --- | --- | --- | --- | --- | --- | --- | --- | --- |
| **Locus in OR960246** | **Locus in U112** | | **Intensity in OR960246** | | **Intensity in U112** | | **Fold Difference** | | **Gene** | **Product** | |
|  |  | |  | |  | |  | |  |  | |
|  |  | |  | |  | |  | |  |  | |
| FTH_1096 | None | | 107.91 | | 1 | | 108 | | FTH_1096 | None | |
| FTH_1243 | None | | 237.63 | | 2.38 | | 100 | | add | None | |
| FTH_1304 | None | | 197.24 | | 6.04 | | 33 | | FTH_1304 | None | |
| FTH_1337 | None | | 135.63 | | 12.92 | | 11 | | FTH_1337 | None | |
| FTH_1464 | None | | 135.06 | | 1.19 | | 114 | | FTH_1464 | None | |
| FTH_1512 | None | | 167.4 | | 8.62 | | 19 | | FTH_1512 | None | |
| FTH_1787 | None | | 167.99 | | 14.07 | | 12 | | FTH_1787 | None | |
| FTH_1788 | None | | 227.92 | | 1.24 | | 184 | | FTH_1788 | None | |
| FTH_1871 | None | | 297.14 | | 3.19 | | 93 | | FTH_1871 | None | |
| FTH_1096 | None | | 107.91 | | 1 | | 108 | | FTH_1096 | None | |
| FTH_1243 | None | | 237.63 | | 2.38 | | 100 | | add | None | |
| FTH_1304 | None | | 197.24 | | 6.04 | | 33 | | FTH_1304 | None | |
| FTH_1337 | None | | 135.63 | | 12.92 | | 11 | | FTH_1337 | | None |
| FTH_1464 | None | | 135.06 | | 1.19 | | 114 | | FTH_1464 | | None |
| FTH_1512 | None | | 167.4 | | 8.62 | | 19 | | FTH_1512 | | None |
| FTH_1787 | None | | 167.99 | | 14.07 | | 12 | | FTH_1787 | | None |
| FTH_1788 | None | | 227.92 | | 1.24 | | 184 | | FTH_1788 | | None |
| FTH_1871 | None | | 297.14 | | 3.19 | | 93 | | FTH_1871 | | None |
|  | |  | |  | |  | |  |  |  | |
