# Supplementary material for: Comparative Transcriptional Analyses of Francisella tularensis and Francisella novicida
Source: PLoS One. 2016 Aug 18;11(8):e0158631. doi: 10.1371/journal.pone.0158631 (PMC4990168; doi:10.1371/journal.pone.0158631)
Supplement: S6 Table — (DOCX) [file pone.0158631.s006.docx]

S6 Table: Genes with High Expression (≥ 5 fold and p≤ 0.05) in *Fn* Compared to *Fth.*

|  |  |  |  |  |  |  | |
| --- | --- | --- | --- | --- | --- | --- | --- |
| **Locus in U112** | **Locus in OR960246** | **Intensity in OR960246** | **Intensity in U112** | **Fold Difference** | **Gene** | **Product** | |
|  |  |  |  |  |  |  | |
|  |  |  |  |  |  |  | |
| **Gene is intact in U112 and its ortholog in OR960246 is also intact** | | | | | | | |
|  | | | | | | | |
| FTN_0022 | FTH_0030 | 75.11 | 504.89 | 7 | FTN_0022 | histidine acid phosphatase | |
| FTN_0033 | FTH_0043 | 41.35 | 573.47 | 14 | FTN_0033 | hypothetical protein | |
| FTN_0034 | FTH_0044 | 99.47 | 466.98 | 5 | FTN_0034 | hypothetical protein | |
| FTN_0060 | FTH_1813 | 13.86 | 659.2 | 48 | leuD | isopropylmalate isomerase small subunit | |
| FTN_0061 | FTH_1814 | 23.57 | 487.47 | 21 | leuC | isopropylmalate isomerase | |
| FTN_0067 | FTH_0125 | 24.12 | 305.75 | 13 | FTN_0067 | hypothetical protein | |
| FTN_0081 | FTH_0164 | 9.71 | 613.74 | 63 | FTN_0081 | hypothetical protein | |
| FTN_0087 | FTH_0155 | 114.64 | 727.05 | 6 | FTN_0087 | allophanate hydrolase subunit 1 | |
| FTN_0094 | FTH_0053 | 47.93 | 303.99 | 6 | FTN_0094 | transcriptional regulator, LysR family | |
| FTN_0100 | FTH_0060 | 49.82 | 560.39 | 11 | FTN_0100 | hypothetical membrane protein | |
| FTN_0113 | FTH_0073 | 261.84 | 1187.62 | 5 | ribC | | riboflavin synthase alpha chain |
| FTN_0114 | FTH_0074 | 179.76 | 1075.3 | 6 | ribD | | pyrimidine reductase |
| FTN_0115 | FTH_0005 | 14.57 | 123.58 | 8 | FTN_0115 | | Na+/H+ antiporter |
| FTN_0128 | FTH_0021 | 48.84 | 466.41 | 10 | FTN_0128 | | metabolite:H+ symporter (MHS) family protein |
| FTN_0148 | FTH_1845 | 34.26 | 686.35 | 20 | FTN_0148 | | hypothetical membrane protein |
| FTN_0343 | FTH_0320 | 38.77 | 508.26 | 13 | FTN_0343 | | aminotransferase |
| FTN_0360 | FTH_0338 | 61.07 | 326.33 | 5 | FTN_0360 | | hypothetical protein |
| FTN_0362 | FTH_0340 | 24.06 | 170.32 | 7 | FTN_0362 | | deoxyribodipyrimidine photolyase-related protein |
| FTN_0471 | FTH_0438 | 85.05 | 1844.26 | 22 | FTN_0471 | | NADPH-dependent FMN reductase |
| FTN_0527 | FTH_0495 | 30.75 | 440.09 | 14 | thrC | | threonine synthase |
| FTN_0532 | FTH_1571 | 41.04 | 192.51 | 5 | FTN_0532 | | hypothetical protein |
| FTN_0533 | FTH_1570 | 65.04 | 365.51 | 6 | FTN_0533 | | drug:H+ antiporter-1 (DHA1) family protein |
| FTN_0588 | FTH_1511 | 70.37 | 773.77 | 11 | FTN_0588 | | asparaginase |
| FTN_0590 | FTH_1509 | 26.06 | 270.86 | 10 | FTN_0590 | | hypothetical protein |
| FTN_0596 | FTH_1501 | 25.9 | 162.21 | 6 | FTN_0596 | | hypothetical protein |
| FTN_0631 | FTH_1456 | 45.95 | 211.33 | 5 | FTN_0631 | | metabolite:H+ symporter (MHS) family protein |
| FTN_0717 | FTH_1332 | 106.27 | 613.92 | 6 | FTN_0717 | | hypothetical protein |
| FTN_0759 | FTH_1290 | 18.29 | 104.96 | 6 | FTN_0759 | | hypothetical protein |
| FTN_0811 | FTH_1249 | 55.45 | 344.76 | 6 | birA | | biotin--acetyl-CoA-carboxylase ligase |
| FTN_0868 | FTH_1189 | 52.67 | 496.38 | 9 | FTN_0868 | | membrane protein |
| FTN_0878 | FTH_1178 | 114.05 | 1436.75 | 13 | FTN_0878 | | hypothetical protein |
| FTN_0884 | FTH_1172 | 49.54 | 379.17 | 8 | FTN_0884 | | drug/metabolite transporter |
| FTN_0973 | FTH_0974 | 100.52 | 2261.07 | 22 | FTN_0973 | | peroxiredoxin |
| FTN_0977 | FTH_0969 | 28.85 | 253.47 | 9 | FTN_0977 | | hypothetical protein |
|  |  |  |  |  |  | |  |

(Continued)

**S6 Table (continued).**

|  |  |  |  |  |  |  | |
| --- | --- | --- | --- | --- | --- | --- | --- |
| **Locus in U112** | **Locus in OR960246** | **Intensity in OR960246** | **Intensity in U112** | **Fold Difference** | **Gene** | **Product** | |
|  |  |  |  |  |  |  | |
|  |  |  |  |  |  |  | |
| FTN_0991 | FTH_0948 | 17.88 | 111.52 | 6 | lldD | L-lactate dehydrogenase | |
| FTN_1044 | FTH_0891 | 233.51 | 1794.71 | 8 | FTN_1044 | hypothetical protein | |
| FTN_1170 | FTH_0754 | 199.04 | 1266.6 | 6 | FTN_1170 | hypothetical protein | |
| FTN_1192 | FTH_1370 | 43.38 | 400.97 | 9 | FTN_1192 | chitin-binding protein | |
| FTN_1252 | FTH_0713 | 15.05 | 302.35 | 20 | FTN_1252 | choloylglycine hydrolase family protein | |
| FTN_1282 | FTH_1169 | 22.42 | 313.95 | 14 | FTN_1282 | transcriptional regulator, LysR family | |
| FTN_1349 | FTH_1104 | 5.28 | 131.03 | 25 | FTN_1349 | hypothetical protein | |
| FTN_1386 | FTH_0642 | 15.55 | 1214.82 | 78 | FTN_1386 | hypothetical protein | |
| FTN_1413 | FTH_0617 | 21.71 | 423.6 | 20 | FTN_1413 | AAA family-ATPase | |
| FTN_1454 | FTH_0565 | 20.61 | 410 | 20 | FTN_1454 | NAD/FAD-binding protein | |
| FTN_1455 | FTH_0563 | 16.41 | 310.4 | 19 | FTN_1455 | | hypothetical protein |
| FTN_1456 | FTH_0562 | 18.79 | 525.07 | 28 | cfa | | cyclopropane fatty acid synthase, methyltransferase |
| FTN_1457 | FTH_0561 | 18.66 | 744.01 | 40 | FTN_1457 | | hypothetical protein |
| FTN_1458 | FTH_0560 | 37.77 | 360.64 | 10 | FTN_1458 | | hypothetical protein |
| FTN_1459 | FTH_0559 | 14.22 | 493.43 | 35 | FTN_1459 | | short chain dehydrogenase |
| FTN_1474 | FTH_0545 | 431.39 | 2372.31 | 5 | bglX | | glycosyl hydrolase family 3 |
| FTN_1511 | FTH_0292 | 155.98 | 908.31 | 6 | FTN_1511 | | hypothetical protein |
| FTN_1529 | FTH_0272 | 39.4 | 341.14 | 9 | FTN_1529 | | glutamate:GABA antiporter (APC family) protein |
| FTN_1530 | FTH_0271 | 35.17 | 270.43 | 8 | lysA | | diaminopimelate decarboxylase |
| FTN_1543 | FTH_1658 | 12.19 | 286.66 | 24 | FTN_1543 | | hypothetical protein |
| FTN_1588 | FTH_1591 | 41.31 | 689.35 | 17 | FTN_1588 | | major facilitator superfamily (MFS) transport protein |
| FTN_1615 | FTH_1699 | 180.91 | 1013.61 | 6 | FTN_1615 | | hypothetical protein |
| FTN_1618 | FTH_1702 | 90.36 | 632.91 | 7 | FTN_1618 | | hypothetical protein |
| FTN_1620 | FTH_1704 | 78.02 | 2316.32 | 30 | appB | | cytochrome bd-II terminal oxidase subunit II |
| FTN_1696 | FTH_1785 | 24.95 | 299.99 | 12 | FTN_1696 | | hypothetical protein |
| FTN_1782 | FTH_1885 | 68.85 | 401.01 | 6 | rng | | ribonuclease G |
|  |  |  |  |  |  | |  |
| **Gene is intact in U112 and its ortholog in OR960246 is a pseudogene** | | | | | | | |
|  |  |  |  |  |  | |  |
| FTN_0003 | FTH_0003 | 23.13 | 513.87 | 22 | FTN_0003 | | metabolite:H+ symporter (MHS) family protein |
| FTN_0032 | FTH_0041 | 20.66 | 807.55 | 39 | FTN_0032 | | hypothetical protein |
| FTN_0057 | FTH_0050 | 25.88 | 306.54 | 12 | FTN_0057 | | major facilitator superfamily (MFS) transport protein |
| FTN_0058 | FTH_0051 | 39.74 | 339.41 | 9 | FTN_0058 | | beta-fructofuranosidase |
| FTN_0059 | FTH_1812 | 7.07 | 605.43 | 86 | leuB | | 3-isopropylmalate dehydrogenase |
|  |  |  |  |  |  | |  |
|  |  |  |  |  |  | |  |

(Continued)

**S6 Table (continued).**

|  |  |  |  |  |  |  | |
| --- | --- | --- | --- | --- | --- | --- | --- |
| **Locus in U112** | **Locus in OR960246** | **Intensity in OR960246** | **Intensity in U112** | **Fold Difference** | **Gene** | **Product** | |
|  |  |  |  |  |  |  | |
|  |  |  |  |  |  |  | |
| FTN_0086 | FTH_0156 | 44.16 | 860.19 | 19 | FTN_0086 | metabolite:H+ symporter | |
| FTN_0093 | FTH_0148 | 91 | 454.65 | 5 | FTN_0093 | cytochrome b561 family protein | |
| FTN_0103 | FTH_0063 | 62.04 | 451.42 | 7 | FTN_0103 | hypothetical protein | |
| FTN_0116 | FTH_0006 | 54.8 | 573.14 | 10 | ipdC | indolepyruvate decarboxylase | |
| FTN_0127 | FTH_0022 | 22.6 | 499.65 | 22 | gabD | succinate semialdehyde dehydrogenase | |
| FTN_0131 | FTH_0127 | 108.26 | 744.9 | 7 | FTN_0131 | hypothetical protein | |
| FTN_0176 | FTH_1848 | 5.89 | 296.59 | 50 | FTN_0176 | serine permease | |
| FTN_0344 | FTH_0321 | 63.82 | 632.22 | 10 | FTN_0344 | aspartate:alanine exchanger (AAE) family protein | |
| FTN_0345 | FTH_0322 | 117.26 | 928.8 | 8 | FTN_0345 | DNA uptake protein, SMF family | |
| FTN_0414 | FTH_0383 | 131.26 | 1305.59 | 10 | FTN_0414 | Type IV pili, pilus assembly protein | |
| FTN_0524 | FTH_0492 | 14.62 | 589.72 | 40 | asd | aspartate semialdehyde dehydrogenase | |
| FTN_0525 | FTH_0493 | 39.23 | 827.15 | 21 | thrA | aspartate kinase I/homoserine dehydrogenase I | |
| FTN_0526 | FTH_0494 | 11.25 | 713.24 | 63 | thrB | homoserine kinase | |
| FTN_0566 | FTH_1534 | 102.02 | 640.4 | 6 | FTN_0566 | ion channel protein | |
| FTN_0579 | FTH_1521 | 45.34 | 563.15 | 12 | FTN_0579 | major facilitator superfamily (MFS) transport protein | |
| FTN_0589 | FTH_1510 | 40.76 | 643.05 | 16 | FTN_0589 | di- or tripeptide:H+ symporter | |
| FTN_0730 | FTH_1319 | 115.37 | 601.77 | 5 | acs | acyl-coenzyme A synthetase/AMP-(fatty) acid ligases | |
| FTN_0740 | FTH_1310 | 25.4 | 480.72 | 19 | FTN_0740 | hypothetical protein | |
| FTN_0747 | FTH_1302 | 177.39 | 1089.98 | 6 | FTN_0747 | | amino acid-polyamine-organocation (APC) superfamily protein |
| FTN_0757 | FTH_1292 | 44.46 | 482.14 | 11 | FTN_0757 | | membrane protein |
| FTN_0767 | FTH_1067 | 38.03 | 201.71 | 5 | betT | | betaine/carnitine/choline transporter (BCCT) family protein |
| FTN_0773 | FTH_1073 | 83.55 | 495.72 | 6 | FTN_0773 | | 4Fe-4S ferredoxin |
| FTN_0776 | FTH_1077 | 15.8 | 130.41 | 8 | FTN_0776 | | DNA and RNA helicases Superfamily I protein |
| FTN_0783 | FTH_1084 | 56.54 | 476.73 | 8 | FTN_0783 | | isochorismatase family protein |
| FTN_0824 | FTH_1235 | 13.17 | 156.5 | 12 | FTN_0824 | | major facilitator superfamily (MFS) transport protein |
| FTN_0827 | FTH_1233 | 50.4 | 342.27 | 7 | FTN_0827 | | carbon-nitrogen |
| FTN_0854 | FTH_1204 | 402.56 | 2283.16 | 6 | FTN_0854 | | hypothetical protein |
| FTN_0872 | FTH_1184 | 89.65 | 437.98 | 5 | FTN_0872 | | small conductance mechanosensitive ion channel (MscS) family protein |
| FTN_0875 | FTH_1181 | 78.25 | 405.66 | 5 | FTN_0875 | | metabolite:H+ symporter |
| FTN_0886 | FTH_1059 | 8.27 | 227.32 | 27 | FTN_0886 | | sugar:cation symporter |
|  |  |  |  |  |  | |  |

(Continued)

**S6 Table (continued).**

|  |  |  |  |  |  |  | |
| --- | --- | --- | --- | --- | --- | --- | --- |
| **Locus in U112** | **Locus in OR960246** | **Intensity in OR960246** | **Intensity in U112** | **Fold Difference** | **Gene** | **Product** | |
|  |  |  |  |  |  |  | |
|  |  |  |  |  |  |  | |
| FTN_0086 | FTH_0156 | 44.16 | 860.19 | 19 | FTN_0086 | metabolite:H+ symporter | |
| FTN_0093 | FTH_0148 | 91 | 454.65 | 5 | FTN_0093 | cytochrome b561 family protein | |
| FTN_0103 | FTH_0063 | 62.04 | 451.42 | 7 | FTN_0103 | hypothetical protein | |
| FTN_0116 | FTH_0006 | 54.8 | 573.14 | 10 | ipdC | indolepyruvate decarboxylase | |
| FTN_0127 | FTH_0022 | 22.6 | 499.65 | 22 | gabD | succinate semialdehyde dehydrogenase | |
| FTN_0131 | FTH_0127 | 108.26 | 744.9 | 7 | FTN_0131 | hypothetical protein | |
| FTN_0176 | FTH_1848 | 5.89 | 296.59 | 50 | FTN_0176 | serine permease | |
| FTN_0344 | FTH_0321 | 63.82 | 632.22 | 10 | FTN_0344 | aspartate:alanine exchanger (AAE) family protein | |
| FTN_0345 | FTH_0322 | 117.26 | 928.8 | 8 | FTN_0345 | DNA uptake protein, SMF family | |
| FTN_0414 | FTH_0383 | 131.26 | 1305.59 | 10 | FTN_0414 | Type IV pili, pilus assembly protein | |
| FTN_0524 | FTH_0492 | 14.62 | 589.72 | 40 | asd | aspartate semialdehyde dehydrogenase | |
| FTN_0525 | FTH_0493 | 39.23 | 827.15 | 21 | thrA | aspartate kinase I/homoserine dehydrogenase I | |
| FTN_0526 | FTH_0494 | 11.25 | 713.24 | 63 | thrB | homoserine kinase | |
| FTN_0566 | FTH_1534 | 102.02 | 640.4 | 6 | FTN_0566 | ion channel protein | |
| FTN_0579 | FTH_1521 | 45.34 | 563.15 | 12 | FTN_0579 | major facilitator superfamily (MFS) transport protein | |
| FTN_0589 | FTH_1510 | 40.76 | 643.05 | 16 | FTN_0589 | di- or tripeptide:H+ symporter | |
| FTN_0730 | FTH_1319 | 115.37 | 601.77 | 5 | acs | acyl-coenzyme A synthetase/AMP-(fatty) acid ligases | |
| FTN_0740 | FTH_1310 | 25.4 | 480.72 | 19 | FTN_0740 | hypothetical protein | |
| FTN_0747 | FTH_1302 | 177.39 | 1089.98 | 6 | FTN_0747 | | amino acid-polyamine-organocation (APC) superfamily protein |
| FTN_0757 | FTH_1292 | 44.46 | 482.14 | 11 | FTN_0757 | | membrane protein |
| FTN_0767 | FTH_1067 | 38.03 | 201.71 | 5 | betT | | betaine/carnitine/choline transporter (BCCT) family protein |
| FTN_0773 | FTH_1073 | 83.55 | 495.72 | 6 | FTN_0773 | | 4Fe-4S ferredoxin |
| FTN_0776 | FTH_1077 | 15.8 | 130.41 | 8 | FTN_0776 | | DNA and RNA helicases Superfamily I protein |
| FTN_0783 | FTH_1084 | 56.54 | 476.73 | 8 | FTN_0783 | | isochorismatase family protein |
| FTN_0824 | FTH_1235 | 13.17 | 156.5 | 12 | FTN_0824 | | major facilitator superfamily (MFS) transport protein |
| FTN_0827 | FTH_1233 | 50.4 | 342.27 | 7 | FTN_0827 | | carbon-nitrogen |
| FTN_0854 | FTH_1204 | 402.56 | 2283.16 | 6 | FTN_0854 | | hypothetical protein |
| FTN_0872 | FTH_1184 | 89.65 | 437.98 | 5 | FTN_0872 | | small conductance mechanosensitive ion channel (MscS) family protein |
| FTN_0875 | FTH_1181 | 78.25 | 405.66 | 5 | FTN_0875 | | metabolite:H+ symporter |
| FTN_0886 | FTH_1059 | 8.27 | 227.32 | 27 | FTN_0886 | | sugar:cation symporter |
|  |  |  |  |  |  | |  |

(Continued)

**S6 Table (continued).**

|  |  |  |  |  |  |  | |
| --- | --- | --- | --- | --- | --- | --- | --- |
| **Locus in U112** | **Locus in OR960246** | **Intensity in OR960246** | **Intensity in U112** | **Fold Difference** | **Gene** | **Product** | |
|  |  |  |  |  |  |  | |
|  |  |  |  |  |  |  | |
| FTN_0898 | FTH_1045 | 12.46 | 166.63 | 13 | FTN_0898 | amino acid permease | |
| FTN_0910 | FTH_1032 | 197.92 | 2112.4 | 11 | FTN_0910 | sugar:cation symporter family protein | |
| FTN_0911 | FTH_1031 | 255.73 | 2100.72 | 8 | FTN_0911 | glycosyl hydrolases family 31 protein | |
| FTN_0926 | FTH_1015 | 152.67 | 711.21 | 5 | cysC | adenylylsulfate kinase | |
| FTN_0962 | FTH_0986 | 34.34 | 1019.62 | 30 | FTN_0962 | hypothetical protein | |
| FTN_0963 | FTH_0985 | 77.94 | 1263.96 | 16 | FTN_0963 | NAD-dependent aldehyde dehydrogenase | |
| FTN_0964 | FTH_0984 | 113.25 | 1780.9 | 16 | FTN_0964 | dehydrogenase | |
| FTN_0969 | FTH_0979 | 13.43 | 224.31 | 17 | FTN_0969 | hypothetical protein | |
| FTN_0972 | FTH_0975 | 13.84 | 181.21 | 13 | FTN_0972 | hypothetical protein | |
| FTN_0976 | FTH_0970 | 9.27 | 718.81 | 78 | FTN_0976 | ThiF family protein | |
| FTN_1010 | FTH_0925 | 7.78 | 230.94 | 30 | FTN_1010 | major facilitator superfamily (MFS) transport protein | |
| FTN_1032 | FTH_0904 | 82.23 | 781.5 | 10 | FTN_1032 | proton-dependent oligopeptide transporter (POT) family protein | |
| FTN_1043 | FTH_0893 | 183.24 | 1224.81 | 7 | ilvD | dihydroxy-acid dehydratase | |
| FTN_1044 | FTH_0890 | 204.77 | 920.31 | 4 | FTN_1044 | hypothetical protein | |
| FTN_1049 | FTH_0885 | 47.83 | 486.23 | 10 | FTN_1049 | oxidoreductase | |
| FTN_1121 | FTH_0811 | 27.31 | 287.36 | 11 | phrB | deoxyribodipyrimidine photolyase | |
| FTN_1143 | FTH_0786 | 129.04 | 1661.77 | 13 | FTN_1143 | 4Fe-4S ferredoxin, FAD dependent | |
| FTN_1152 | FTH_0776 | 30.44 | 163.32 | 5 | FTN_1152 | methyltransferase | |
| FTN_1155 | FTH_0771 | 5.51 | 169.54 | 31 | FTN_1155 | type I restriction-modification system, subunit R (restriction) | |
| FTN_1169 | FTH_0756 | 12.07 | 327.87 | 27 | FTN_1169 | peptidase, M20 family | |
| FTN_1186 | FTH_0737 | 93.03 | 4427.41 | 48 | pepO | M13 family metallopeptidase | |
| FTN_1251 | FTH_0714 | 129.93 | 669.89 | 5 | FTN_1251 | proton-dependent oligopeptide transporter (POT) family protein, di- or tripeptide:H+ symporter | |
| FTN_1261 | FTH_0704 | 51.21 | 2672.69 | 52 | FTN_1261 | hypothetical protein | |
| FTN_1280 | FTH_0685 | 32.14 | 596.88 | 19 | FTN_1280 | tryptophan repressor binding protein-like flavidoxin | |
| FTN_1325 | FTH_1128 | 128.82 | 1173.93 | 9 | pdpD | hypothetical protein | |
| FTN_1347 | FTH_1106 | 85.09 | 406.6 | 5 | sun | tRNA and rRNA cytosine-C5-methylases, sun protein | |
| FTN_1385 | FTH_0643 | 31.25 | 671.32 | 21 | FTN_1385 | hypothetical protein | |
| FTN_1391 | FTH_0637 | 33.56 | 174.39 | 5 | naoX | uncharacterized NAD(FAD)-dependent dehydrogenase | |
| FTN_1445 | FTH_0577 | 59.96 | 1213.31 | 20 | FTN_1445 | hypothetical protein | |
| FTN_1453 | FTH_0567 | 60.25 | 381.71 | 6 | FTN_1453 | two-component regulator, sensor histidine kinase | |
|  |  |  |  |  |  | |  |

(Continued)

**S6 Table (continued).**

|  |  |  |  |  |  |  | |
| --- | --- | --- | --- | --- | --- | --- | --- |
| **Locus in U112** | **Locus in OR960246** | **Intensity in OR960246** | **Intensity in U112** | **Fold Difference** | **Gene** | **Product** | |
|  |  |  |  |  |  |  | |
|  |  |  |  |  |  |  | |
| FTN_1460 | FTH_0558 | 45.11 | 547.2 | 12 | FTN_1460 | delta 9 acyl-lipid fatty acid desaturase | |
| FTN_1466 | FTH_0552 | 35.69 | 419.79 | 12 | FTN_1466 | hypothetical protein | |
| FTN_1531 | FTH_0270 | 29.53 | 167.7 | 6 | FTN_1531 | hypothetical protein | |
| FTN_1533 | FTH_1265 | 4.5 | 322.07 | 72 | FTN_1533 | hypothetical protein | |
| FTN_1589 | FTH_1592 | 4.62 | 313.9 | 68 | oppF | PepT family protein | |
| FTN_1590 | FTH_1592 | 27.56 | 1146.59 | 42 | oppD | PepT family protein | |
| FTN_1591 | FTH_1593 | 42.94 | 849.97 | 20 | oppC | PepT family protein | |
| FTN_1592 | FTH_1594 | 34.84 | 996.49 | 29 | oppB | PepT family protein | |
| FTN_1592 | FTH_1594 | 13.07 | 1053.34 | 81 | oppB | PepT family protein | |
| FTN_1593 | FTH_1595 | 62.16 | 901.23 | 14 | oppA | ABC-type oligopeptide transport system, periplasmic component | |
| FTN_1616 | FTH_1700 | 58.24 | 830.7 | 14 | FTN_1616 | hypothetical protein | |
| FTN_1616 | FTH_1700 | 27.48 | 502.68 | 18 | FTN_1616 | hypothetical protein | |
| FTN_1619 | FTH_1703 | 30.73 | 1833.9 | 60 | appC | cytochrome bd-II terminal oxidase subunit I | |
| FTN_1621 | FTH_1706 | 27 | 1361.59 | 50 | FTN_1621 | oxidoreductase | |
| FTN_1692 | FTH_1779 | 63.61 | 491.85 | 8 | FTN_1692 | membrane fusion protein | |
| FTN_1693 | FTH_1780 | 95.05 | 537.05 | 6 | FTN_1693 | ATP-binding cassette (ABC) superfamily protein | |
| FTN_1708 | FTH_1798 | 19.39 | 121.7 | 6 | FTN_1708 | ATP-binding cassette (ABC) superfamily protein | |
| FTN_1714 | FTH_1804 | 95.5 | 644.67 | 7 | kdpE | two-component response regulator | |
| FTN_1715 | FTH_1805 | 134.26 | 757.47 | 6 | kdpD | two component regulator, sensor histidine kinase kdpD | |
| FTN_1719 | FTH_1809 | 110.31 | 766.04 | 7 | FTN_1719 | D-isomer specific 2-hydroxyacid dehydrogenase | |
| FTN_1733 | FTH_0099 | 29.17 | 430.02 | 15 | FTN_1733 | nicotinamide ribonucleoside (NR) uptake permease (PnuC) family protein | |
| FTN_1755 | FTH_0077 | 107.29 | 511.68 | 5 | FTN_1755 | metabolite:H+ symporter (MHS) family protein | |
|  |  |  |  |  |  |  | |
| **Gene is intact in U112 and its ortholog in OR960246 is absent** | | | | | | | |
|  |  |  |  |  |  |  | |
| FTN_0004 | None | 1.43 | 646.69 | 453 | FTN_0004 | aspartate/glutamate transporter | |
| FTN_0005 | None | 1 | 357.82 | 358 | corA | divalent inorganic cation transporter | |
| FTN_0006 | None | 1 | 385.09 | 385 | FTN_0006 | hypothetical protein | |
| FTN_0007 | None | 1.91 | 1859.31 | 975 | FTN_0007 | hypothetical protein | |
| FTN_0008 | None | 2.21 | 719.31 | 326 | FTN_0008 | TMS drug/metabolite exporter protein | |
| FTN_0017 | None | 17.69 | 119.18 | 7 | FTN_0017 | phage integrase | |
|  |  |  |  |  |  | |  |

(Continued)

**S6 Table (continued).**

|  |  |  |  |  |  |  |
| --- | --- | --- | --- | --- | --- | --- |
| **Locus in U112** | **Locus in OR960246** | **Intensity in OR960246** | **Intensity in U112** | **Fold Difference** | **Gene** | **Product** |
|  |  |  |  |  |  |  |
|  |  |  |  |  |  |  |
| FTN_0025 | None | 1.19 | 303.86 | 256 | FTN_0025 | hypothetical protein |
| FTN_0038 | None | 1.53 | 128.22 | 84 | FTN_0038 | hypothetical protein |
| FTN_0039 | None | 7.46 | 435.64 | 58 | FTN_0039 | hypothetical protein |
| FTN_0040 | None | 1.12 | 339.52 | 304 | FTN_0040 | hypothetical protein |
| FTN_0041 | None | 1.03 | 440.74 | 428 | FTN_0041 | hypothetical protein |
| FTN_0042 | None | 1 | 378.08 | 378 | FTN_0042 | hypothetical protein |
| FTN_0043 | None | 19.33 | 604.07 | 31 | FTN_0043 | hypothetical protein |
| FTN_0044 | None | 9.42 | 1140.76 | 121 | FTN_0044 | hypothetical protein |
| FTN_0045 | None | 3.33 | 353.35 | 106 | FTN_0045 | hypothetical protein |
| FTN_0046 | None | 10.34 | 407.95 | 39 | FTN_0046 | hypothetical protein |
| FTN_0047 | None | 2.04 | 363.85 | 179 | FTN_0047 | hypothetical protein |
| FTN_0048 | None | 1.58 | 408.31 | 258 | FTN_0048 | hypothetical protein |
| FTN_0049 | None | 4.72 | 408.92 | 87 | FTN_0049 | hypothetical protein |
| FTN_0050 | None | 8.01 | 441.99 | 55 | FTN_0050 | hypothetical protein |
| FTN_0051 | None | 8.94 | 1059.99 | 119 | FTN_0051 | hypothetical protein |
| FTN_0052 | None | 2.27 | 451.66 | 199 | FTN_0052 | hypothetical protein |
| FTN_0054 | None | 1.1 | 530.19 | 481 | FTN_0054 | hypothetical protein |
| FTN_0062 | None | 8.94 | 443.08 | 50 | leuA | 2-isopropylmalate synthase |
| FTN_0115 | None | 3.15 | 104.74 | 33 | FTN_0115 | Na+/H+ antiporter |
| FTN_0130 | None | 13.56 | 309.59 | 23 | FTN_0130 | glycosyl transferase, group 1 |
| FTN_0150 | None | 1.24 | 736.76 | 595 | FTN_0150 | YggT family protein |
| FTN_0153 | None | 2.88 | 125.28 | 43 | FTN_0153 | RimI-like acetyltransferase |
| FTN_0154 | None | 7.54 | 176.09 | 23 | rimK | glutathione synthase/ribosomal protein S6 modification enzyme |
| FTN_0215 | None | 1 | 149.15 | 149 | FTN_0215 | hypothetical protein |
| FTN_0267 | None | 1 | 670.02 | 670 | FTN_0267 | hypothetical protein |
| FTN_0282 | None | 1.31 | 647.13 | 495 | FTN_0282 | hypothetical protein |
| FTN_0287 | None | 2.39 | 102.99 | 43 | FTN_0287 | type I restriction-modification system, subunit R (restriction) |
| FTN_0308 | None | 1.41 | 212.43 | 151 | FTN_0308 | membrane protein |
| FTN_0309 | None | 6.29 | 323.76 | 51 | FTN_0309 | hypothetical membrane protein |
| FTN_0358 | None | 6.82 | 633.52 | 93 | FTN_0358 | tRNA-methylthiotransferase |
| FTN_0359 | None | 14.67 | 681.06 | 46 | FTN_0359 | short-chain alcohol dehydrogenase |
| FTN_0369 | None | 6.21 | 311.36 | 50 | FTN_0369 | hypothetical protein |
| FTN_0370 | None | 1.47 | 479.7 | 326 | FTN_0370 | hypothetical protein |
| FTN_0371 | None | 13.42 | 179.27 | 13 | FTN_0371 | hypothetical protein |
| FTN_0415 | None | 1 | 3254.27 | 3254 | pilA | Type IV pili, pilus assembly protein |
| FTN_0418 | None | 1.62 | 809.72 | 501 | FTN_0418 | endonuclease |
| FTN_0451 | None | 1.51 | 847.72 | 563 | FTN_0451 | signal transduction protein |
| FTN_0452 | None | 14.69 | 284.75 | 19 | FTN_0452 | hypothetical protein |
| FTN_0453 | None | 10.47 | 138.01 | 13 | FTN_0453 | glycosyl transferase |
| FTN_0454 | None | 3.4 | 135.46 | 40 | FTN_0454 | hypothetical protein |
|  |  |  |  |  |  |  |
|  |  |  |  |  |  |  |

(Continued)

**S6 Table (continued).**

|  |  |  |  |  |  |  |
| --- | --- | --- | --- | --- | --- | --- |
| **Locus in U112** | **Locus in OR960246** | **Intensity in OR960246** | **Intensity in U112** | **Fold Difference** | **Gene** | **Product** |
|  |  |  |  |  |  |  |
|  |  |  |  |  |  |  |
| FTN_0455 | None | 1.41 | 583.03 | 413 | FTN_0455 | CheB methylesterase |
| FTN_0456 | None | 2.24 | 339.3 | 151 | FTN_0456 | signal transduction |
| FTN_0509 | None | 8.87 | 308.72 | 35 | FTN_0509 | hypothetical protein |
| FTN_0510 | None | 1.52 | 192.69 | 127 | FTN_0510 | hypothetical protein |
| FTN_0525 | None | 5.69 | 833.23 | 146 | thrA | aspartate kinase I/homoserine dehydrogenase I |
| FTN_0526 | None | 4 | 446.97 | 112 | thrB | homoserine kinase |
| FTN_0578 | None | 1.97 | 269.39 | 137 | FTN_0578 | major facilitator superfamily (MFS) transport protein |
| FTN_0587 | None | 3.56 | 424.34 | 119 | FTN_0587 | deoxyguanosinetriphosphate triphosphohydrolase |
| FTN_0617 | None | 2.78 | 915.86 | 330 | FTN_0617 | ROK family protein |
| FTN_0618 | None | 8.61 | 826.51 | 96 | FTN_0618 | ROK family protein |
| FTN_0627 | None | 1.06 | 391.29 | 369 | chiA | chitinase |
| FTN_0703 | None | 4.07 | 173.53 | 43 | FTN_0703 | type I restriction-modification system, subunit S |
| FTN_0704 | None | 2.22 | 277.72 | 125 | FTN_0704 | methyltransferase |
| FTN_0705 | None | 3.78 | 262.39 | 69 | FTN_0705 | abortive infection bacteriophage resistance protein |
| FTN_0707 | None | 1.56 | 369.69 | 236 | FTN_0707 | type I restriction-modification system, subunit S |
| FTN_0708 | None | 4.23 | 245.52 | 58 | FTN_0708 | hypothetical protein |
| FTN_0709 | None | 1 | 146.88 | 147 | FTN_0709 | hypothetical protein |
| FTN_0710 | None | 2.59 | 302.03 | 116 | FTN_0710 | type I restriction-modification system, subunit R (restriction) |
| FTN_0711 | None | 9.82 | 270.9 | 28 | FTN_0711 | metal-dependent hydrolase |
| FTN_0730 | None | 1.6 | 631.99 | 394 | acs | acyl-coenzyme A synthetase |
| FTN_0757 | None | 1 | 513.11 | 513 | FTN_0757 | membrane protein |
| FTN_0761 | None | 5.64 | 999.64 | 177 | FTN_0761 | radical SAM family protein |
| FTN_0762 | None | 1 | 899.46 | 899 | grxC | glutaredoxin like protein |
| FTN_0763 | None | 6.03 | 680.05 | 113 | FTN_0763 | thioesterase superfamily protein |
| FTN_0776 | None | 19.89 | 1106.69 | 56 | FTN_0776 | DNA and RNA helicases Superfamily I protein |
| FTN_0825 | None | 6.58 | 852.11 | 130 | FTN_0825 | aldo/keto reductase family protein |
| FTN_0837 | None | 2.74 | 353.36 | 129 | FTN_0837 | hypothetical protein |
| FTN_0839 | None | 24.55 | 2225.26 | 91 | FTN_0839 | hypothetical protein |
| FTN_0840 | None | 55.44 | 1951.63 | 35 | mdaB | NADPH-quinone reductase |
| FTN_0841 | None | 7.68 | 2074.96 | 270 | FTN_0841 | ThiJ/PfpI family protein |
| FTN_0885 | None | 7.79 | 567.4 | 73 | FTN_0885 | di- or tripeptide:H+ symporter |
| FTN_0928 | None | 1.72 | 239.18 | 139 | cysD | sulfate adenylyltransferase subunit 2 |
| FTN_0929 | None | 4.53 | 257.74 | 57 | FTN_0929 | hypothetical protein |
| FTN_0930 | None | 28.13 | 559.64 | 20 | FTN_0930 | hypothetical protein |
| FTN_0931 | None | 1.57 | 428.27 | 273 | FTN_0931 | hypothetical protein |
|  |  |  |  |  |  |  |

(Continued)

**S6 Table (continued).**

|  |  |  |  |  |  |  |
| --- | --- | --- | --- | --- | --- | --- |
| **Locus in U112** | **Locus in OR960246** | **Intensity in OR960246** | **Intensity in U112** | **Fold Difference** | **Gene** | **Product** |
|  |  |  |  |  |  |  |
|  |  |  |  |  |  |  |
| FTN_0932 | None | 1.03 | 331.23 | 321 | FTN_0932 | ATP-binding protein |
| FTN_0933 | None | 1.44 | 365.07 | 254 | FTN_0933 | hypothetical protein |
| FTN_0934 | None | 1.26 | 386.06 | 305 | FTN_0934 | hypothetical protein |
| FTN_0935 | None | 3.18 | 260.5 | 82 | asnB | asparagine synthase |
| FTN_0936 | None | 1 | 182.44 | 182 | FTN_0936 | hypothetical protein |
| FTN_0937 | None | 1.75 | 206.31 | 118 | FTN_0937 | hypothetical protein |
| FTN_0938 | None | 1.07 | 914.79 | 855 | FTN_0938 | hypothetical protein |
| FTN_0939 | None | 1.14 | 1760.31 | 1549 | FTN_0939 | hypothetical protein |
| FTN_0962 | None | 10.87 | 1885.84 | 173 | FTN_0962 | hypothetical protein |
| FTN_0963 | None | 23.72 | 1510.03 | 64 | FTN_0963 | aldehyde dehydrogenase |
| FTN_0965 | None | 1.21 | 238.99 | 198 | FTN_0965 | metal-dependent exopeptidase |
| FTN_0984 | None | 6.68 | 249.86 | 37 | FTN_0984 | ABC transporter |
| FTN_0998 | None | 29.12 | 694.82 | 24 | FTN_0998 | potassium channel protein |
| FTN_1043 | None | 12.57 | 1631.06 | 130 | ilvD | dihydroxy-acid dehydratase |
| FTN_1079 | None | 23.38 | 798.5 | 34 | FTN_1079 | sugar porter (SP) family |
| FTN_1101 | None | 10.12 | 290.04 | 29 | FTN_1101 | hypothetical protein |
| FTN_1102 | None | 1.04 | 336.49 | 323 | FTN_1102 | hypothetical protein |
| FTN_1103 | None | 1.33 | 523.53 | 394 | FTN_1103 | hypothetical protein |
| FTN_1104 | None | 13.57 | 660.25 | 49 | FTN_1104 | hypothetical protein |
| FTN_1012 | None | 65.65 | 383.92 | 6 | FTN_1012 | mechanosensitive ion channel family protein |
| FTN_1083 | None | 66.03 | 343.46 | 5 | FTN_1083 | hypothetical protein |
| FTN_1153 | None | 1.16 | 155.99 | 134 | FTN_1153 | hypothetical protein |
| FTN_1154 | None | 2.6 | 285.76 | 110 | FTN_1154 | type I restriction-modification system, subunit S |
| FTN_1215 | None | 1.46 | 696.92 | 477 | kpsC | capsule polysaccharide export protein KpsC |
| FTN_1216 | None | 3.2 | 563.62 | 176 | FTN_1216 | hypothetical protein |
| FTN_1230 | None | 29.41 | 647.7 | 22 | FTN_1230 | hypothetical protein |
| FTN_1260 | None | 9.43 | 323.45 | 34 | FTN_1260 | hypothetical membrane protein |
| FTN_1261 | None | 2.41 | 2648.04 | 1097 | FTN_1261 | hypothetical protein |
| FTN_1281 | None | 3.67 | 138.91 | 38 | FTN_1281 | pirin family protein |
| FTN_1325 | None | 36.62 | 1128.33 | 31 | pdpD | hypothetical protein |
| FTN_1326 | None | 58.87 | 798.57 | 14 | FTN_1326 | hypothetical protein |
| FTN_1378 | None | 1 | 401.29 | 401 | FTN_1378 | hypothetical protein |
| FTN_1397 | None | 18.86 | 426.06 | 23 | FTN_1397 | hypothetical protein |
| FTN_1420 | None | 11.16 | 813.2 | 73 | wzx | O antigen flippase |
| FTN_1422 | None | 1.78 | 1352.75 | 759 | wbtN | glycosyl transferase, group 1 |
| FTN_1424 | None | 1.16 | 621.34 | 537 | FTN_1424 | hypothetical membrane protein |
| FTN_1427 | None | 11.06 | 1831.48 | 166 | wbtD | glycosyl transferase, group 1 |
| FTN_1428 | None | 2.37 | 2229.52 | 940 | wbtO | transferase |
| FTN_1429 | None | 4 | 1841.02 | 460 | wbtP | galactosyl transferase |
| FTN_1430 | None | 15.63 | 2067.64 | 132 | wbtQ | aminotransferase |
|  |  |  |  |  |  |  |

(Continued)

**S6 Table (continued).**

|  |  | |  | |  | |  | |  |  | |
| --- | --- | --- | --- | --- | --- | --- | --- | --- | --- | --- | --- |
| **Locus in U112** | **Locus in OR960246** | | **Intensity in OR960246** | | **Intensity in U112** | | **Fold Difference** | | **Gene** | **Product** | |
|  |  | |  | |  | |  | |  |  | |
|  |  | |  | |  | |  | |  |  | |
| FTN_1432 | None | | 1.85 | | 184.44 | | 100 | | hrpA | HrpA-like helicase | |
| FTN_1454 | None | | 1.77 | | 223.14 | | 126 | | FTN_1454 | NAD/FAD-binding protein | |
| FTN_1487 | None | | 2.97 | | 265.88 | | 90 | | FTN_1487 | restriction endonuclease | |
| FTN_1488 | None | | 5.96 | | 465.08 | | 78 | | FTN_1488 | prophage maintenance system killer protein (DOC) | |
| FTN_1489 | None | | 1.82 | | 534.72 | | 294 | | FTN_1489 | hypothetical protein | |
| FTN_1490 | None | | 2.11 | | 337.84 | | 160 | | FTN_1490 | hypothetical protein | |
| FTN_1491 | None | | 11.6 | | 598.95 | | 52 | | FTN_1491 | adenine specific DNA methylase | |
| FTN_1589 | None | | 3.4 | | 1017.2 | | 299 | | oppF | peptide/opine/nickel uptake transporter (PepT) family protein | |
| FTN_1590 | None | | 15 | | 1099.13 | | 73 | | oppD | peptide/opine/nickel uptake transporter (PepT) family protein | |
| FTN_1698 | None | | 5.4 | | 1147.9 | | 212 | | FTN_1698 | Dam-replacing family protein | |
| FTN_1727 | None | | 12.08 | | 155.97 | | 13 | | dapD | tetrahydrodipicolinate succinylase subunit | |
| FTN_1728 | None | | 11.85 | | 103.63 | | 9 | | dapA | dihydrodipicolinate synthase | |
| FTN_1729 | None | | 2.19 | | 136.88 | | 63 | | dapB | dihydrodipicolinate reductase | |
| FTN_1730 | None | | 6.7 | | 219.04 | | 33 | | lysC | aspartate kinase III | |
| FTN_1755 | None | | 5.89 | | 377.85 | | 64 | | FTN_1755 | metabolite:H+ symporter (MHS) family protein | |
| FTN_1756 | None | | 1.71 | | 578.58 | | 337 | | bcp | bacterioferritin comigratory protein | |
| FTN_1757 | None | | 3.52 | | 540.56 | | 154 | | FTN_1757 | 2-hydroxyacid dehydrogenase | |
| FTN_1759 | None | | 4.41 | | 409.83 | | 93 | | FTN_1759 | hypothetical protein | |
| FTN_1760 | None | | 20.27 | | 129.79 | | 6 | | FTN_1760 | zinc-binding alcohol dehydrogenase | |
| FTN_1766 | None | | 2.01 | | 583.36 | | 290 | | FTN_1766 | drug/metabolite transporter (DMT) superfamily protein | |
| FTN_1767 | None | | 10.77 | | 582.51 | | 54 | | rbsK | ribokinase, pfkB family | |
|  |  | |  | |  | |  | |  |  | |
| **Gene is pseudogene in U112 and its ortholog in OR960246 is intact** | | | | | | | | | | | |
|  |  | |  | |  | |  | |  |  | |
| FTN_0784 | FTH_1086 | | 156.4 | | 1554.66 | | 10 | | FTN_0784 | None | |
|  |  | |  | |  | |  | |  |  | |
| **Gene is pseudogene in U112 and its ortholog in OR960246 is also a pseudogene** | | | | | | | | | | | |
|  |  | |  | |  | |  | |  |  | |
| FTN_1614 | FTH_1698 | | 46.61 | | 354.88 | | 8 | | FTN_1614 | None | |
|  |  | |  | |  | |  | |  |  | |
| **Gene is pseudogene in U112 and its ortholog in OR960246 is absent** | | | | | | | | | | | |
|  |  | |  | |  | |  | |  |  | |
| FTN_0283 | None | | 3.07 | | 169.11 | | 55 | | FTN_0283 | None | |
| FTN_1379 | None | | 3.25 | | 197.02 | | 61 | | FTN_1379 | None | |
|  | |  | |  | |  | |  |  |  |  |
