# Supplementary material for: Comparative Transcriptional Analyses of Francisella tularensis and Francisella novicida
Source: PLoS One. 2016 Aug 18;11(8):e0158631. doi: 10.1371/journal.pone.0158631 (PMC4990168; doi:10.1371/journal.pone.0158631)
Supplement: S7 Table — (DOCX) [file pone.0158631.s007.docx]

S7 Table: Expression of Transposases

|  |  |  |  |  |
| --- | --- | --- | --- | --- |
| **Transposase** | **Intensity in LVS** | **Intensity in OR960246** | **Intensity in Schu S4** | **Intensity in U112** |
|  |  |  |  |  |
|  |  |  |  |  |
| isftu1 | 3242.22 | 3857.92 | 3235.64 | 97.11 |
| isftu3 | 158.26 | 135.94 | 138.49 | 49.46 |
| isftu4 | 9.08 | 21.16 | 24.6 | 1 |
| isftu5 | 55.34 | 54.96 | 73.16 | 2.34 |
| isftu6 | 177.09 | 334.09 | 143.81 | 3.51 |
|  |  |  |  |  |
